# Genetic Regulatory Perturbation of Gene Expression Impacted by Genomic Introgression in Fiber Development of Allotetraploid Cotton

**DOI:** 10.1002/advs.202401549

**Published:** 2024-08-28

**Authors:** Xinyuan Chen, Xiubao Hu, Guo Li, Corrinne E. Grover, Jiaqi You, Ruipeng Wang, Zhenping Liu, Zhengyang Qi, Xuanxuan Luo, Yabin Peng, Mengmeng Zhu, Yuqi Zhang, Sifan Lu, Yuan‐ming Zhang, Zhongxu Lin, Jonathan F. Wendel, Xianlong Zhang, Maojun Wang

**Affiliations:** ^1^ National Key Laboratory of Crop Genetic Improvement, Hubei Hongshan Laboratory Huazhong Agricultural University Wuhan 430070 China; ^2^ Crop Information Center, College of Plant Science and Technology Huazhong Agricultural University Wuhan 430070 China; ^3^ Department of Ecology, Evolution, and Organismal Biology Iowa State University Ames IA 50011 USA

**Keywords:** cotton fibers, eQTL, genetic regulation, introgression, population transcriptomics

## Abstract

Interspecific genomic introgression is an important evolutionary process with respect to the generation of novel phenotypic diversity and adaptation. A key question is how gene flow perturbs gene expression networks and regulatory interactions. Here, an introgression population of two species of allopolyploid cotton (*Gossypium*) to delineate the regulatory perturbations of gene expression regarding fiber development accompanying fiber quality change is utilized. *De novo* assembly of the recipient parent (*G. hirsutum* Emian22) genome allowed the identification of genomic variation and introgression segments (ISs) in 323 introgression lines (ILs) from the donor parent (*G. barbadense* 3–79). It documented gene expression dynamics by sequencing 1,284 transcriptomes of developing fibers and characterized genetic regulatory perturbations mediated by genomic introgression using a multi‐locus model. Introgression of individual homoeologous genes exhibiting extreme low or high expression bias can lead to a parallel expression bias in their non‐introgressed duplicates, implying a shared yet divergent regulatory fate of duplicated genes following allopolyploidy. Additionally, the IL N182 with improved fiber quality is characterized, and the candidate gene *GhFLAP1* related to fiber length is validated. This study outlines a framework for understanding introgression‐mediated regulatory perturbations in polyploids, and provides insights for targeted breeding of superior upland cotton fiber.

## Graphical abstract

This work studies the genetic regulatory perturbation of gene expression of the recipient genome impacted by introgression in plants, with regulatory effects on the formation of agronomic traits. The significance of genomic introgression in phenotypic innovation is discussed from the dimensions of variation, regulation, and polyploidization. These findings provide valuable perspectives and resources for cotton breeding efforts.

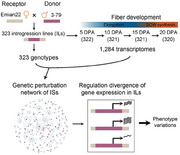

## 1 Introduction

Interspecific gene flow, or introgression, is an important process shaping genetic diversity within and among species^[^ ^1^, ^2^, ^3^, ^4^ ^]^ that has been consequential in crop evolution.^[^ ^5^, ^6^, ^7^, ^8^, ^9^ ^]^ This is increasingly evident using genome sequencing datasets in numerous plant species, where introgression has been shown to increase genetic diversity and play an important role in adaptation and phenotypic innovation.^[^ ^10^, ^11^, ^12^, ^13^ ^]^ In plant breeding, there is a long history of crop scientists using wild species and genetically related gene pools to improve modern cultivated species, and in recent decades this has entailed using advanced mapping populations, such as introgression lines^[^ ^14^, ^15^, ^16^ ^]^ and nested association mapping (NAM) populations.^[^ ^17^, ^18^ ^]^ In major crops such as rice, maize, wheat, and tomato, multiple loci associated with phenotypic variation have been identified using introgression lines.^[^ ^19^, ^20^, ^21^, ^22^, ^23^ ^]^ While the phenomenon and importance of interspecific introgression are widely appreciated, less is understood regarding the genetic mechanisms that generate the resulting phenotypic variation.^[^ ^20^ ^]^ In this respect, the development of expression quantitative trait locus (eQTL) maps has provided a powerful tool for dissecting the complex genetic basis of expression variation and establishing the link between expression and phenotype.^[^ ^24^, ^25^, ^26^, ^27^ ^]^

Cotton (*Gossypium* spp.) is grown widely throughout the world and produces the most important natural fiber in the textile industry. Improving fiber quality is thus an important objective in cotton breeding. The utilization of natural populations has broad advantages for discovering genetic loci related to fiber traits,^[^ ^8^, ^28^, ^29^, ^30^ ^]^ and our recent studies also have integrated eQTL analysis to map the regulatory network related to fiber development.^[^ ^26^, ^31^ ^]^ The development of advanced mapping populations provides another approach for exploring trait‐related loci and expanding genetic resources for cotton breeding.^[^ ^32^, ^33^ ^]^ This is exemplified through the use of introgression line populations of different varieties to shape fiber phenotypes.^[^ ^34^, ^35^ ^]^ The introgression lines, which involve the introgression of segments from *G. barbadense* into the genome of *G. hirsutum*, have made significant contributions to studying the major agronomic traits of cotton.^[^ ^34^, ^36^, ^37^, ^38^ ^]^ Multiple loci associated with fiber length, fiber strength and other traits have been successfully identified. It is worth noting that although introgression line populations have been widely used in crops, the accuracy of existing methods for mapping genetic loci from exogenous genomic segments in populations remains suboptimal in that they typically do not fully define the effect of introgression segments on phenotype.^[^ ^39^ ^]^ Finally, there is limited understanding of how introgression segments alter regulatory networks genome‐wide.^[^ ^40^ ^]^

**Figure 1:**
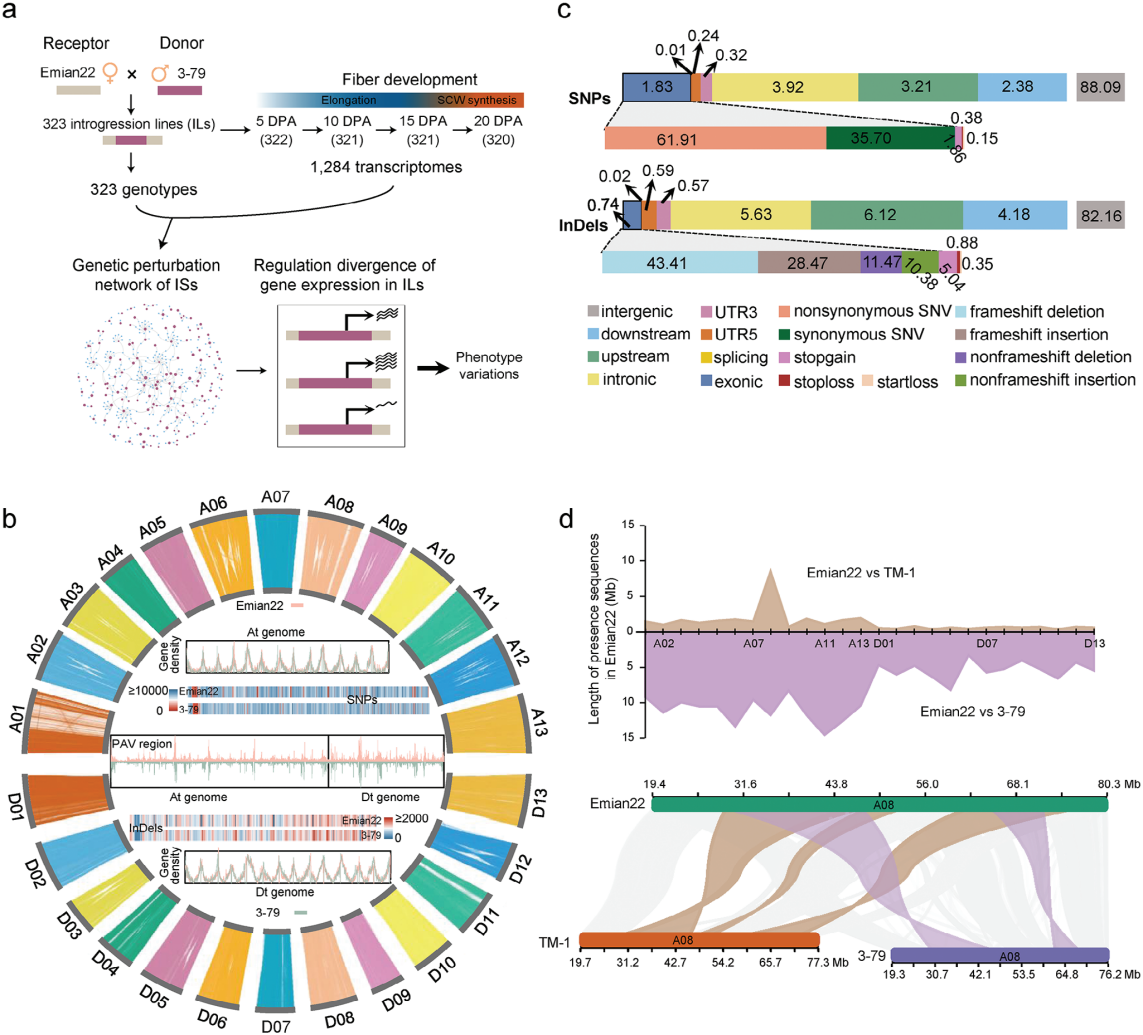
Genomic variation between Emian22 and 3–79. a) Graphic of sampling timepoints, datasets generated and collected, as well as the workflow in the study. Segments originating from 3–79 are depicted in purple, while segments from Emian22 are represented in light khaki. SCW: secondary cell wall. b) Genomic variation map of Emian22 and 3–79. The circle shows the genome‐wide alignment information of Emian22 and 3–79. The inner tracks of the circle represent 3–79, and the outer tracks represent Emian22. Included in the circle: gene density in the A ubgenome (At) and D  subgenome (Dt); SNPs and InDels density between Emian22 and 3–79, where the first heatmap bar shows Emian22 and the second heatmap bar shows 3–79; and PAV density in the At and Dt. Pink indicates Emian22, and green indicates 3–79. All these data are shown in 1 megabase pairs (Mb) windows sliding 200 kilobase pairs (Kb). c) The annotation of SNPs and InDels between Emian22 and 3–79. The upper part of the stacked histogram represents the variation regions of SNPs and InDels. The lower part of the histogram represents the functional annotation of the variant in the exon region. The numbers in the stacked histogram represent the percentage of each block. d) Genomic differences between Emian22 and TM‐1/3‐79. The upper panel describes the length of unique sequences in Emian22 identified between Emian22 and TM‐1/3‐79 on each chromosome. The lower panel shows the alignment information between Emian22 and TM‐1/3‐79 in a specific region of chromosome A08.

In our previous study, we constructed an introgression line population between *G. hirsutum* Emian22, a high‐yield cultivar with moderate fiber quality, and *G. barbadense* 3–79, a genetic standard with superior fiber.^[^ ^35^, ^41^ ^]^ Introgression between these two species is of great interest due to their complementary properties (i.e., yield and fiber quality) whose genetic underpinnings may be elucidated by evaluating introgression populations. To explore regulatory perturbations of gene expression caused by introgression, we generated a genome assembly for the recipient (Emian22) genome of the IL population to improve the accuracy of genetic analysis, and performed direct genomic comparisons with the donor parent (3‐79).^[^ ^41^ ^]^ We generated 1284 fiber transcriptome datasets for developing cotton fiber samples from 323 different introgression lines using 4 fiber developmental timepoints (5 days post‐anthesis (DPA), 10 DPA, 15 DPA and 20 DPA). These timepoints encompass primary cell wall synthesis and the transition to secondary wall synthesis. We developed a multi‐locus model method to estimate additive and dominant effects simultaneously, to identify genetic associations between introgression segments and gene expression, as well as with fiber quality‐related traits. This study thus presents a comprehensive depiction of genome‐wide regulatory perturbations in gene expression caused by interspecific introgression, a topic of general interest with respect to speciation, but also of importance to agronomic traits in a major crop (**Figure** **1** **a**).

## 2 Results

### 2.1 Genomic Variation Map between *G. hirsutum* Emian22 and *G. barbadense* 3–79

We *de novo* assembled the genome of *G. hirsutum* Emian22, resulting in a final genome assembly of 2.24 gigabase pairs (Gb), with a contig N50 of 11.14 Mb and scaffold N50 of 97.54 Mb (Tables S1–S6 and Figure S1, Supporting Information). About 99.94% of the 2.24 Gb assembled genome was oriented and organized into 26 pseudochromosomes (Table S1, Supporting Information). Benchmarking Universal Single‐Copy Orthologue (BUSCO, 98.70% of the 1614 core eukaryotic genes) analysis indicated the high completeness and contiguity of the assembled genome (Tables S1 and S7, Supporting Information). The assessment of the LTR Assembly Index (LAI) and the assembly of centromeres also suggest high completeness, contiguity, and correctness of the Emian22 genome (Tables S1, S8 and S9, Supporting Information). We predicted a total of 76102 protein‐coding genes in the Emian22 genome, 95.02% of which were subjected to functional annotation (Tables S10 and S11, Supporting Information).

To identify genomic variations, we performed a comparative genomic analysis between Emian22 and the donor parent 3–79 (Figure 1b,c; Figures S2 and S3, Tables S12–S17, Supporting Information). A total of 11808 genes were predicted to have changes in protein sequences caused by codon loss or gain and frameshifts (Figure 1c; Tables S12 and S13, Supporting Information). Simultaneously, we identified 126.8 Mb of inversion sequences between Emian22 and 3–79, including 93.2 Mb from At and 33.5 Mb from Dt (Table S14, Supporting Information). Approximately 2447 regions were translocated, including 9.1 Mb interchromosomal and 6.2 Mb intrachromosomal translocations (Table S15, Supporting Information). The unique sequences, amounting to 213 Mb in Emian22 and 165 Mb in 3–79, were identified through presence/absence variation (PAV) analysis (Tables S16 and S17, Supporting Information).

By comparing Emian22 with *G. hirsutum* TM‐1, we identified 58.7 Mb inversions and 36.9 Mb translocations (Figures S4 and S5, Supporting Information). PAV analysis revealed that the length of unique sequences in Emian22 was 34.42 Mb, representing only 16% of the difference between Emian22 and *G. barbadense* 3–79 (Figure 1d). These findings indicate that the genomic differences within *G. hirsutum* are significantly smaller than those between *G. hirsutum* and *G. barbadense*. Nevertheless, we observed notable rearrangements within *G. hirsutum*, such as an inversion on chromosome A08 (Figure 1d). The differences among *G. hirsutum* accessions highlight the importance of the assembly of the Emian22 genome in precisely identifying sequence variations in used introgression line population employed here.

We used the genome assemblies and resequencing data of 323 introgression lines to identify introgression segments from *G. barbadense* 3–79.^[^ ^35^, ^41^ ^]^ We identified 1217 introgression segments with a total length of 1908.62 Mb, covering 85.43% of the Emian22 genome, including 88.49% of At and 80.09% of Dt (Figures S6–S8, Tables S18 and S19, Supporting Information). The low coverage of introgression observed on chromosomes A07, D11, D12 and D13 may be related to the frequency of recombination events in this region,^[^ ^6^ ^]^ or possibly to selection against recombinants. The number of introgression segments in each IL ranged from 1 to 21, and the length ranged from 0.05 Mb to 104.61 Mb (Tables S18 and S20, Supporting Information), of which ≈30% had lengths less than 500 kb and 30% greater than 2 Mb (Figure S9, Supporting Information). In particular, 63217 genes were found in introgression segments (Table S19, Supporting Information). Characterization of introgression segments in the introgression population provides a resource for fine mapping complex traits and identifying causative genes.

### 2.2 Dynamics of Gene Expression in the Introgression Lines

**Figure 2:**
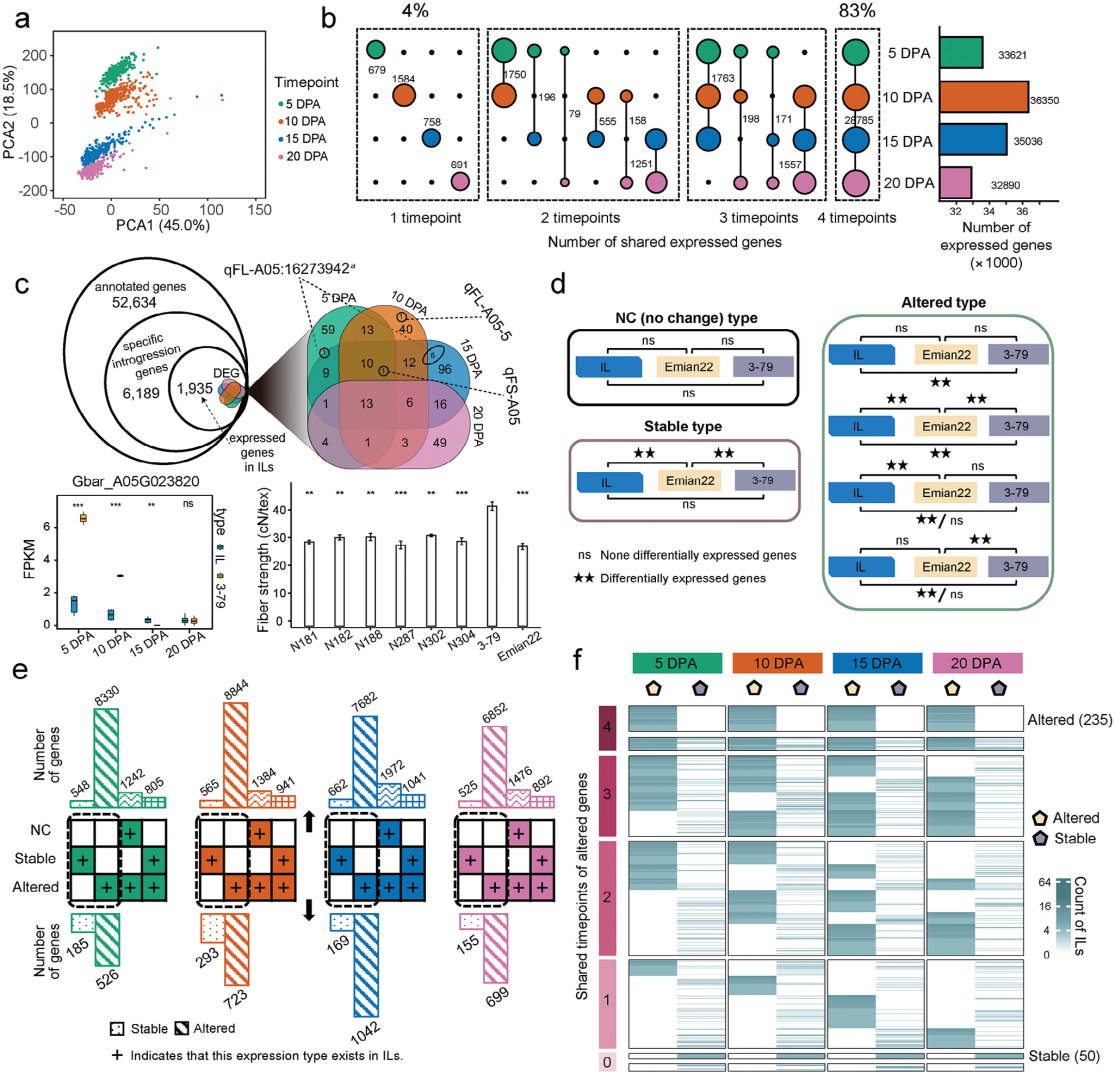
Differential expression patterns of IL population transcriptomes. a) PCA of the transcriptome at four timepoints of fiber development in ILs. b) Characteristic distribution of expressed genes in the transcriptome of populations in 4 timepoints. The size of the circles represents the number of shared expressed genes. The bar graph shows the number of expressed genes at four timepoints. The expressed gene show genes whose expression levels > 1 in at least 3 ILs in the population. c) Characterization of differentially expressed genes specifically annotated in 3–79. The annotated genes describe genes derived from introgression region of 3–79. The specific introgression genes show only genes annotated in 3–79. The expressed gene show genes whose expression levels > 1 in at least 3 ILs in the population. The DEGs show differentially expressed genes between ILs and parent 3–79. The Venn plot shows the distribution of the number of differentially expressed genes in the four timepoints. The number in the black circle represents the number of genes located in this QTL, and the position of the black circle represents the timepoints at which it is differentially expressed between the introgression line and 3–79. Boxplot describes gene (Gbar_A05G023820) expression differences between ILs (n = 6) and 3–79 (n = 3) at 4 timepoints. The histogram describes the difference in fiber strength between the ILs carrying the QTL: *qFS‐A05* and the parent 3–79, n = 5. Significance is tested by two‐tailed Student's *t* test. ns, *P* > 0.05; *, *P* < 0.05; **, *P* < 0.01; ***, *P* < 0.001. Center line, median; box limits, first and third quartiles; whisker, 1.5× interquartile range. Error bars are presented as mean ± SE. d) Defining patterns of the variation degree of differentially expressed genes in a population. e) Statistics of the number of differentially expressed genes in three patterns at four developmental timepoints. The upper panel displays all differentially expressed genes, and the lower panel displays differentially expressed genes that appeared in one type by adding filter conditions for non‐introgressed segments. Colored squares with “+” represent the presence of this expression pattern, and blanks represent the absence. Dotted bars indicate that only the stable type was exhibited in the introgression line population for these genes. Slashed bars indicate that these genes exhibit only the altered type in the introgression line population. The bar with wavy lines indicates that these genes exhibit no change and altered types in the introgression line population. Crosshatched bar indicate that these genes exhibit both stable and altered types in the introgression line population. f) Cluster heatmap showing the distribution of the altered and stable gene expression types in the population for four timepoints. Heatmap colors represent the number of introgression lines. The heatmap was divided into 4 regions according to the shared timepoints of altered genes (red squares). In all cases, green represents 5 DPA, orange represents 10 DPA, blue represents 15 DPA, and purple represents 20 DPA.

To understand the potential genetic effect of introgression segments on fiber development, we characterized gene expression in all 323 introgression lines. We generated a total of 1284 fiber transcriptomes sampled at 4 fiber developmental timepoints (5, 10, 15 and 20 DPA) (with 8 failing to yield samples), which averaged 40 million read pairs per sample. These data were mapped to the Emian22 reference genome (Table S21, Supporting Information). Principal component analysis (PCA) of transcriptome data showed that samples from the same timepoint clustered together (**Figure** **2**a), especially for data from 5 DPA and 10 DPA and data from 15 DPA and 20 DPA, reflecting the fact that the two earlier timepoints sample two stages of primary cell elongation whereas the latter two represent the transition to secondary wall synthesis. We examined the expression of 33621 genes at 5 DPA, 36350 genes at 10 DPA, 35036 genes at 15 DPA, and 32890 genes at 20 DPA. Approximately 83% of all expressed genes were shared among the 4 timepoints, and few (4%) were expressed at only one timepoint (Figure 2b).

We also used the expression data to examine alterations in gene expression between homologs in the IL and the 3–79 parent for those genes exclusively annotated in 3–79 due to the presence of unique regions (such as presence/absence variations) in that genome. We first reconstructed the positions of the introgression segments in the 3–79 genome, which included 52634 annotated genes (Figure 2c; Table S22, Supporting Information). Nearly 12% of these (6189 genes) were identified only in the 3–79 parent, of which only 332 (5.3%) exhibited expression differences between the IL copy and its respective 3–79 homolog (Figure 2c; Table S23, Supporting Information). These genes highlight, and to a certain extent pinpoint, regulatory differences between the two species during fiber development.

After excluding genes annotated only in 3–79, we identified differentially expressed genes between ILs and the two parents across the 4 timepoints. In all introgression segments, more genes exhibited differential expression relative to the Emian22 parent (2470, 2806, 3504 and 2856 genes at 5, 10, 15, and 20 DPA, respectively) versus the 3–79 parent (2122, 2343, 2376 and 1971 genes) (Figure S10, Supporting Information), as may be expected considering 3–79 represents the source of the introgression; however, this difference is perhaps smaller than expected, possibly due to *trans* regulation of these introgressed genes by other native Emian22 genes. Interestingly, differential expression was abundant in non‐introgressed regions as well, where 9135, 20148, 12934 and 11752 differentially expressed genes (relative to the Emian22 parent) were identified at each timepoint (Figure S10, Supporting Information), likely reflecting additional *trans‐*interactions between the Emian22 background and the 3–79 introgression segments as a factor in gene expression.

We classified the expression patterns of all genes from introgression regions into three types, i.e., 1) genes with no expression differences from their parents, or “no change”; 2) genes in the introgression segments that exhibited the same expression as 3–79, or “stable” expression; and 3) genes in the introgression segments that exhibited expression changes, or “altered” expression, as shown in Figure 2d and Figure S11 (Supporting Information). We screened genes of the stable and altered types by adding filter conditions for non‐introgressed segments and retained genes showing only stable or altered expression. The number of genes considered “altered” was 3.9 times (2422/617) that of the stable type for all four timepoints (Figure 2e). When we consider these across the timepoints surveyed, cluster analysis reveals that only 285 genes (9.36%, n = 2991) exhibit the same expression patterns among the four timepoints (Figure 2f), implying the specificity of gene expression patterns in different timepoints. Functional enrichment analysis showed that compared with stably expressed genes, altered genes were enriched in some specific biological processes related to fiber development, such as hemicellulose metabolism, flavonoid biosynthesis and cytoskeleton regulation (Figure S12, Supporting Information), pinpointing functional implications in fiber developmental processes.

### 2.3 Genetic Perturbation of Introgression Segments

Genetic variation may shape phenotypic diversity by regulating gene expression.^[^ ^20^ ^]^ To investigate the regulatory roles of genetic variation from 3–79 relative to Emian22 in transcriptional alterations, we used introgression segments and transcriptome data to perform expression QTL (eQTL) analysis. A total of 1217 introgression segments were partitioned into 1448 nonoverlapping segments using the principle of minimum overlap to reveal genetic impacts of each introgressed locus (Figure S13 and Table S24, Supporting Information).

Given that previously applied methods of linkage analysis are unable to estimate dominance effects in an introgression line population,^[^ ^39^ ^]^ we developed a multi‐locus model method to estimate additive and dominant effects simultaneously (see Methods). Using the 1448 introgression segments divided above, a Monte Carlo simulation study was carried out to investigate the performance of the new method. All the simulated datasets were analyzed by the new method. The average powers of additive and dominance QTL detection were 67.16% and 32.24%, respectively (Table S25, Supporting Information). The false‐positive rate was 5.59‰. These results suggest that this new method has greatly improved efficiency in identifying QTLs relative to previous methods,^[^ ^39^ ^]^ especially in the detection of heterozygous genotypes.

**Figure 3:**
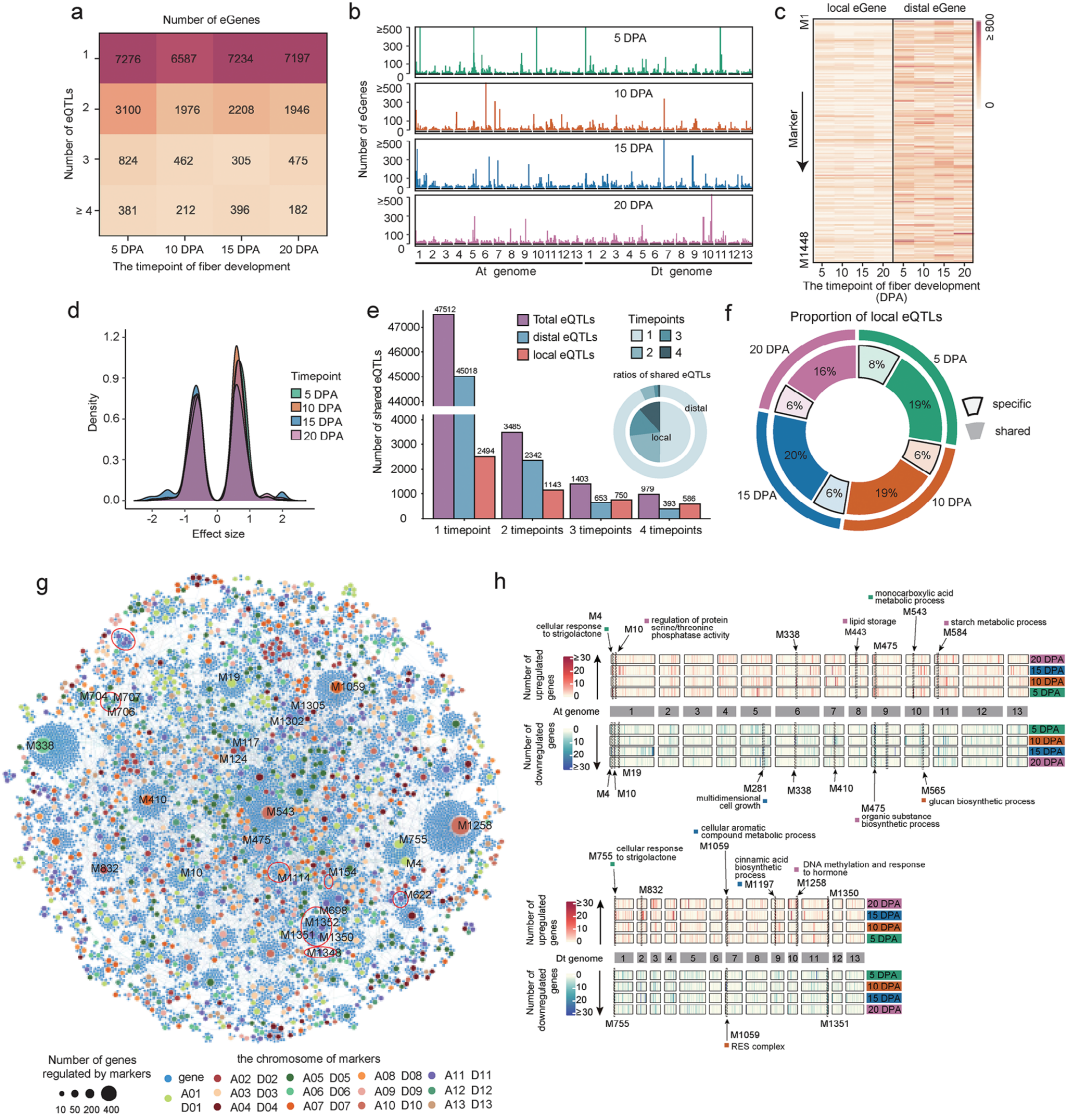
Regulatory perturbation of the Emian22 genome by introgression segments. a) Statistics of eQTLs and eGenes at 4 timepoints. b) The number of eGenes regulated by each marker in the genome. c) Heatmap depicting numbers of local and distal eGenes regulated by markers. d) Density plot of eQTL regulatory effect strength. e) Number of total eQTLs, local eQTLs and distal eQTLs shared over time. The bar chart provides an overview of the shared eQTLs across different timepoints. The x‐axis represents the number of shared timepoints, and the y‐axis represents the number of shared eQTLs. The pie chart describes the ratios of shared eQTLs for local eQTLs and distal eQTLs at 4 timepoints. f) Number of shared and specific distributions of local eQTLs at four timepoints. g) Network of the Emian22 genome showing regulatory perturbation by introgression segments. Blue dots represent the eGenes regulated by the eQTLs, with dots of different colors representing the positions of the markers on the chromosome, and the size of the dot representing the number of eGenes regulated by the marker. h) Distribution of the number of differentially expressed genes in each eQTL. Up‐ and down‐regulated genes are shown symmetrically in the top and bottom panels, respectively. The middle represents the chromosome where each eQTL is located. The number of differentially expressed genes is displayed in a heatmap, where red represents the number of up‐regulated genes and blue represents the number of down‐regulated genes. The small squares of different colors at the bottom of each arrow indicate that this eQTL has the highest number of differentially expressed genes in that period. Text next to each square describes the main enriched functions of these differentially expressed genes. Each eQTL enriched function satisfies false discovery rate (*FDR*) < 0.05.

A total of 17761, 12878, 15493 and 13441 eQTLs (with significance thresholds of logarithm of odds (LOD) ≥ 4) were identified at the four timepoints (Figure S14a, Supporting Information). According to the relative distance between eQTLs and genes, all eQTLs were divided into 4987 local eQTLs (≤1 Mb) and 50897 distal eQTLs (> 1 Mb or on different chromosomes) (Figure S14b, Supporting Information). A total of 1149 expression genes (eGenes) were regulated by local eQTLs, 21656 eGenes were regulated by distal eQTLs, and 3152 eGenes were regulated by both (Figure S14a, Supporting Information). We found that 11101 (42.77%) eGenes were regulated by a single eQTL (Figure S14b, Supporting Information). However, at least 60% of the eGenes were observed to be regulated by only a single eQTL in each period, suggesting that changes in the regulatory relationships may complicate gene transcriptional regulation in fiber development (**Figure** **3**a). We identified 80 eQTL hotspots on different chromosomes at the 4 timepoints (Figure 3b). The number of genes regulated by local eQTLs was significantly smaller than that by distal eQTLs at each timepoint (Figure 3c).

We assessed the sharing and specificity of eQTLs among developmental timepoints using the effect sizes of eQTLs (Figure 3d). We found that 89% of the eQTLs were specific to one timepoint, and 979 eQTLs were shared among the 4 timepoints (Figure 3e). Furthermore, in comparison to local eQTLs, distal eQTLs tend to be more prevalent at each timepoint (Figure 3e). We examined the distribution of local eQTLs and found that the proportion of shared local eQTLs detected in at least two timepoints was approximately twice that of specific local eQTLs (Figure 3e and f). Considering these observations and previous eQTL studies,^[^ ^42^ ^]^ we hypothesize that distal eQTLs may have an important role in generating temporal specificity of gene expression.

By integrating differentially expressed genes and eQTLs, regulatory relationships between introgression segments and genes in each IL were established (Figure 3g; Table S26, Supporting Information). We observed that 75% of the genes in the genetic perturbation network were regulated by distal eQTLs (Figure 3g; Figure S15, Supporting Information). Analysis of the gene types regulated by eQTLs showed that both the number of eGenes in the two subgenomes and the direction of regulation were similar (Figure S15, Supporting Information). Following that, we dissected the distribution of expression changes and number of eGenes regulated by each eQTL (Figure 3h). Most of the eQTL hotspots were largely specific to one time point, representing the accumulation of differentially expressed genes in fiber development. These results highlight the temporal dynamics of genetic perturbations accompanying biological process changes during fiber development. EQTL M1258 exhibited the largest gene expression changes, with ≈98% of genes upregulated at 20 DPA (Figure 3h). These genes were enriched in DNA methylation and hormone response‐related pathways (such as jasmonic acid, auxin and gibberellin) (Figure 3h), suggesting that this eQTL may affect the later developmental process of fibers.

### 2.4 Introgression and Extreme Expression

**Figure 4:**
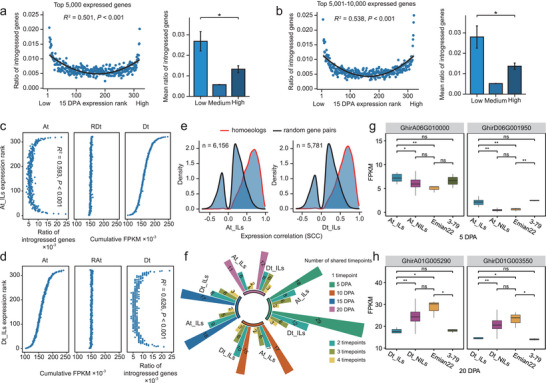
Coordinated expression regulation of homoeologous genes. a–e) both represent analyses performed at 15 DPA. a and b) show the relationship between the expression rank and introgressed genes. Quadratic regression at 15 DPA is shown here (n  =  323 ILs). Each point is the ratio of introgressed genes for lines that share an expression rank for one of the 5000 genes. c and d) show the effect of introgression on homoeologous gene pairs. a) Left panel, significant quadratic relationship between the expression rank of each line for each of the top 5000 most‐expressed genes and the ratio of introgressed genes. Right panel, comparison of the mean ratio of introgressed genes for individuals in the bottom expression ranks (n  =  16) versus the middle two quartiles (n  =  161) versus the top expression ranks (n  =  16) within the top 5000 most‐expressed genes. Error bars are presented as mean ± SE. b) Left panel, significant quadratic relationship between the expression rank of each line in each of the medium 5000–10 000 most‐expressed genes and the ratio of introgressed genes. Right panel, comparison of the mean ratio of introgressed genes for individuals in the bottom expression ranks (n  =  16) versus the middle two quartiles (n  =  161) versus the top expression ranks (n  =  16) within the next 5000 most‐expressed genes. Error bars are presented as mean ± SE. c) The distribution of the ratio of introgressed genes, the cumulative FPKM of the non‐introgressed Dt copy in the random gene pair, and the cumulative FPKM of the non‐introgressed Dt copy in the homoeologous gene pair for the expression rank of the homoeologous gene pair At introgression copy. RDt: the non‐introgressed Dt copy in the random gene pair. At_ILs: introgression of At copy in homoeologous gene pairs. d) The distribution of the ratio of introgressed genes, the cumulative FPKM of the non‐introgressed At copy in the random gene pair, and the cumulative FPKM of the non‐introgressed At copy in the homoeologous gene pair for the expression rank of the homoeologous gene pair Dt introgression copy. RAt: the non‐introgressed At copy in the random gene pair. Dt_ILs: introgression of Dt copy in homoeologous gene pairs. e) SCC density plots of random and homoeologous gene pairs in At_ILs and Dt_ILs. f) The number of homoeologous gene pairs whose non‐introgressed copy is regulated by the introgressed copy at the four timepoints. g) Differences in the expression of At introgressed copies and Dt non‐introgressed copies in At_ILs. h) Differences in the expression of Dt introgressed copies and At non‐introgressed copies in Dt_ILs. In all cases, significance is tested by two‐tailed Student's *t* test. ns, *P* > 0.05; *, *P* < 0.05; **, *P* < 0.01; ***, *P* < 0.001; ****, *P* < 0.0001. ILs: introgression accessions; NILs: non‐introgression accessions. Center line, median; box limits, first and third quartiles; whisker, 1.5× interquartile range.

Recent genetic analyses in both plants and animals have shown that rare variants may cause phenotypically relevant dysregulation of expression.^[^ ^43^, ^44^, ^45^, ^46^ ^]^ To explore if there is evidence for this phenomenon in cotton, we investigated the relationship between the proportion of introgressed genes and their expression levels at each timepoint. Specifically, the 5000 genes with the highest expression level were ranked, and the average number of introgressed genes in the 5000 gene combinations was plotted. The proportion of introgressed genes was correlated with the number of extremely expressed genes (R^2^ = 0.501 and *P* < 0.001; **Figure** **4**a,b; Figure S16, Supporting Information). Although the quadratic regression was highly significant, more extreme trends were observed in the tails of the regression curves, so we analyzed the enrichment of introgressed genes in the 5% expression ranks at both ends. Of note is the observation that the number of introgressed genes was enriched 3.53‐4.88‐fold in the 16 introgression lines with the lowest expression rank and 1.90‐2.77‐fold in the 16 introgression lines with the highest expression rank compared with the middle expression rank (the middle two quartiles) (Figure 4a,b; Figure S16, Supporting Information). These data indicate that extreme expression was biased toward extremely low expression.

### 2.5 Genetic Regulation of Homoeologous Gene Pairs

Because allopolyploid cotton contains duplicates of the majority of genes in the genome (At and Dt), and because they may share *cis*‐regulatory sequences due to common ancestry,^[^ ^47^, ^48^ ^]^ it is of interest to explore homoeologous gene expression in the presence of introgression of only one of the two homoeologs. At 5 DPA, the number of differentially expressed homoeologous genes between the introgressed copy and its homoeolog from Emian22 was 1431, a number that is 2.5 times as high as that for non‐introgressed homoeologous duplicates (n = 577) (Figure S17, Supporting Information). A total of 373 genes in the random non‐introgressed copy had expression differences from the parents, but the number of differentially expressed genes in the homoeologous non‐introgressed copy was ≈1.5 times that of the random non‐introgressed copy, suggesting that the homoeologous introgression may have an effect on expression of homoeologous non‐introgressed copies (Figure S17, Supporting Information). We observed similar trends in other timepoints (Figure S17, Supporting Information).

To better analyze the expression changes of homoeologous genes after introgression, we compared the expression of non‐introgressed copies in homoeologous gene pairs and random gene pairs. The results showed that extreme expression of homoeologous introgressed copies may alter gene expression of its homoeologous non‐introgressed copy (Figure 4c,d; Figure S18, Supporting Information). These results imply a common regulatory system that coordinates transcriptional regulation of homoeologous gene copies.

In an effort to quantify the expression synergism for a subset of homoeologous genes, the expression correlation coefficients of homoeologous genes and random gene pairs were calculated. We observed that the distribution of expression correlation coefficients for homoeologous gene pairs was strongly shifted toward positive values compared with random gene pairs, demonstrating a positive regulatory effect on expression between homoeologous genes (Figure 4e; Figure S19, Supporting Information). eQTL–eGene association explains the effect of genetic variation on gene expression, which also partially explains the phenomenon of coordinated expression of homoeologous gene pairs. A shared eQTL was defined as an eQTL that regulates both At and Dt homoeologs. From the eQTL results, 1000 shared eQTLs were identified in homoeologous gene pairs with At introgression, but only 48 shared eQTLs were identified in random gene pairs with At introgression (Figure S20a, Supporting Information). In the case of Dt introgression, we also observed that the number of shared eQTLs for homoeologous genes was 30 times higher than that in random gene pairs (Figure S20a, Supporting Information). We analyzed the number of eQTLs at the four timepoints and found 265 eQTL–eGene (introgressed copy – non‐introgressed copy) associations in homoeologous gene pairs (Figure 4f; Figure S20b, Supporting Information). Analysis of these homoeologous genes showed that the differential expression patterns of the introgressed copies among samples was similar to that of the corresponding non‐introgressed copies (Figure 4g and h). These results suggest that coordinated regulation of homoeologous gene pairs may be related to shared eQTLs or that one homoeologous copy serves as the regulatory sequence for the other copy.

### 2.6 Effect of Introgression Segments on Fiber Quality

**Figure 5:**
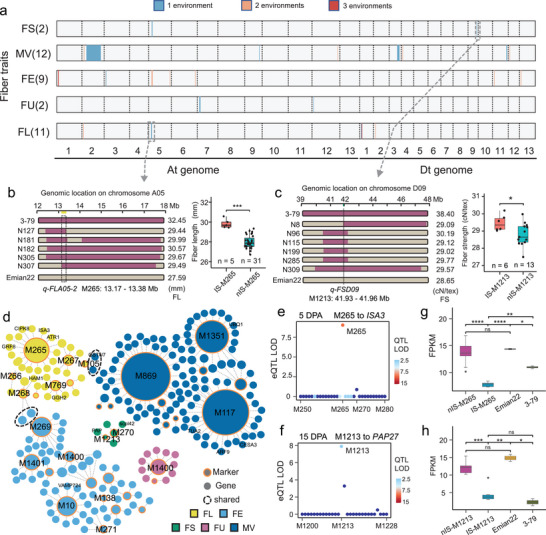
Genetic basis and regulation divergence of introgression segments in Emian22. a) Genome distribution of significant QTLs related to fiber quality. Different colors represent the number of environments in which the QTL was found. b) An example of an FL‐related marker. Left panel, fiber length values and introgression segment location for lines containing M265. Right panel, box plot showing the comparison of fiber length between accessions that carry M265‐IS (n = 5) and those that do not carry M265‐IS (n = 31). c) An example of an FS‐related marker. Left panel, fiber length values and introgression segment location for lines containing M1213. Right panel, box plot showing the comparison of fiber strength between accessions that carry M1213‐IS (n = 6) and those that do not carry M1213‐IS (n = 13). d) Co‐localization results for each of 5 fiber traits. A circle with a yellow edge indicates a marker; otherwise, it is a gene. Dashed circles represent shared genes or markers. e) Co‐localization plots for the M265 locus, where the QTL signal (heatmap) and eQTL signal (x‐axis) reached the significance threshold. Heatmap shows LOD values of QTLs. f) Co‐localization plots for the M1213 locus, where the QTL signal (heatmap) and eQTL signal (x‐axis) reached the significance threshold. Heatmap shows LOD values of QTLs. g) Differences in the expression of genes regulated by M265 in accessions that carry M265‐IS (n = 5) and those that do not carry M265‐IS (n = 31), as well as the parents (Emian22, n = 3; 3–79, n = 3). h) Differences in expression of genes regulated by M1213 accessions that carry M1213‐IS (n = 6) and those that do not carry M1213‐IS (n = 13), as well as the parents (Emian22, n = 3; 3–79, n = 3). IS‐M265: accessions that carry M265‐IS, nIS‐M265: accessions that do not carry M265‐IS. IS‐M1213: accessions that carry M1213‐IS, nIS‐M1213: accessions that do not carry M1213‐IS. In all cases, significance is tested by two‐tailed Student's *t* test. ns, *P* > 0.05; *, *P* < 0.05; **, *P* < 0.01; ***, *P* < 0.001; ****, *P* < 0.0001. Center line, median; box limits, first and third quartiles; whisker, 1.5× interquartile range.

To identify genomic segments favorably associated with fiber quality, we identified quantitative trait locus (QTLs) using the re‐sequencing and phenotypic data from 323 introgression lines (Table S27, Supporting Information).^[^ ^35^, ^41^ ^]^ A total of 36 QTLs were identified, of which 11 were associated with fiber length (FL), 2 with fiber strength (FS), 12 with micronaire value (MV), 9 with fiber elongation rate (FE), and 2 with fiber uniformity (FU) (**Figure** **5**a; Table S28, Supporting Information). Among them, 16 QTLs were newly identified. Notable among these was the fiber length QTL *q‐FLA05‐2*, which was contained within an introgression segment (13.17–13.38 Mb) on chromosome A05. The fiber length of ILs carrying this introgression segment was greater than that of the recipient parent Emian22 and ILs without introgression (Student's *t* test; *P* < 0.001; Figure 5b). Also notable was the fiber strength QTL *q‐FSD09* identified on chromosome D09; this introgression segment can increase fiber strength (Student's *t* test; *P* < 0.05; Figure 5c).

We mapped the QTL results to the 3–79 genome and found that 8 differentially expressed genes from the 3–79 unique regions were located within the identified QTLs. Seven genes were found to be located in the reported QTL *qFL‐A05:16 273 942* (Figure 2c; Table S23, Supporting Information). The gene Gbar_A05G023820, which was differentially expressed in 3–79 at 5 DPA, 10 DPA and 15 DPA, was mapped to *qFS‐A05*. Phenotype analysis revealed that the fiber strength of ILs containing this gene was significantly lower than that of 3–79 (Student's *t* test; *P* < 0.01 and *P* < 0.001; Figure 2c), implying that the unique sequences from 3–79 may also be regulated by the Emian22 genome, thereby affecting the fiber phenotype.

We next used eQTL data to pinpoint candidate genes responsible for these fiber quality‐related QTLs. We found that 34 loci colocalized with eQTLs for the genes associated with this fiber trait (Figure 5d; Table S29, Supporting Information). The candidate gene *ISA3* at *q‐FLA05‐2* encodes an isoamylase‐like protein, which appears regulated by eQTL M265 at 5 DPA (Figure 5e) and exhibits significantly different expression between ILs with and without the introgression segment (Student's *t* test; *P* < 0.0001; Figure 5g). Moreover, the expression of the gene in the introgression segment was also significantly different from that of the parents (Student's *t* test; *P* < 0.0001 and *P* < 0.01; Figure 5g), implying that its inherited expression from the parent 3–79 was affected by the genome of Emian22. This, however, was not true for all genes within introgression segments; for example, expression of *PAP27* (purple acid phosphatase 27), located in an introgression segment and regulated by M1213, was not significantly different from 3–79 but was significantly different from both non‐introgressed segment ILs and Emian22 (Student's *t* test: *P* > 0.05, *P* < 0.001 and *P* < 0.01; Figure 5f and h), suggesting that the differences in expression of this gene may be primarily specified within that segment. Considering these different outcomes, we characterized changes in expression of co‐localized genes after the introgression of 3–79 segments. Approximately 40 genes retained the same expression patterns as 3–79 after segment introgression; however, introgression resulted in increased expression (relative to Emian22) for 13 genes and decreased expression for 18 genes (Figure 1a). These data indicate the potential use of *G. barbadense* genomic segments in fiber quality improvement of *G. hirsutum*, and considerations.

### 2.7 Differential Genetic Regulation Mediated by Different Introgression Segments

Our established framework of localized genetic perturbations provides an excellent opportunity to evaluate the pyramiding effect of introgression segments on phenotypic changes. We selected single‐chromosome segment introgression lines to characterize the regulatory and phenotypic diversity associated with different introgression segments. By comparing fiber quality between the single‐segment IL N69 and the parents, we found significant differences in micronaire value (Student's *t* test; *P* < 0.05; Figure S21a, Supporting Information). Confirming this observation, micronaire values were lower in carrying N69‐IS lines (n = 6) than in without N69‐IS lines (n = 55) (Student's *t* test; *P* < 0.05; Figure S21b and c, Supporting Information).

We observed that IL N101, which carries a partial introgression segment relative to N69, had a micronaire value similar to the parental Emian22 that was statistically different from IL N69 (Student's *t* test; *P* <0.05; Figure S21a and b, Supporting Information). In contrast, another introgression line IL N178 also carried a partial introgression segment relative to N69, yet it exhibited no difference in micronaire value from IL N69 (Student's *t* test; *P* > 0.05; Figure S21a, b and d, Supporting Information). After refining the introgression segment of N69 (divided into 3 markers: M1350, M1351 and M1352), we find that N178 (M1350, M1351) contained a longer segment than N101 (M1351, M1352) (Figure S21b and d, Supporting Information). Interestingly, we found the three markers were identified as QTLs related to micronaire value (Figure S21d and Table S28, Supporting Information).

The impact of different combinations of introgression segments on the fiber phenotype prompts us to consider whether there are differences in genetic regulation between these combinations. We delineated the genetic perturbation networks of the introgression segments in the introgression lines containing N69‐IS from an established genetic framework (Figure S21e and f, Supporting Information). The number of genes regulated by M1350 (n = 23) was 10 times greater than the number of genes regulated by M1352 (n = 2). Interestingly, when the introgression segment on chromosome D11 was longer than the introgression segment from N69 IL, there was no difference in micronaire value between these ILs and N69 (Figure S21a and b, Supporting Information). We speculate that the additional sequence introduced in these longer introgression lines affected the genes regulated by the narrower N69 introgression segment. We screened the genetic perturbation network for introgression lines that overlap the N69 introgression segment, and found that introgression lines with longer introgression tracks on D11 also regulate the expression of genes whose expression is regulated by N69‐IS (Figure S21d,e and f, Supporting Information). These findings indicate that different combinations of introgression segments gave rise to the formation of distinct regulatory relationships in the introgression lines (Figure S21d,e and f, Supporting Information).

Another case, we observed a set of five QTLs associated with fiber length on chromosome A05 (Figure S22a and b, Table S28, Supporting Information). We obtained a similar finding to the N69‐IS study by examining the fiber phenotype and genetic regulatory relationships in ILs containing these sequences (5 QTL related to fiber length) (Figure S22, Supporting Information). Collectively, these results highlight the potential of different combinations of introgression segments to alter genetic regulatory networks, and thereby impact fiber quality.

### 2.8 Comparison between Ancient‐ISs and IL‐ISs

Although *G. hirsutum* and *G. barbadense* arose on different continents as wild species (Central America and South America, respectively) and were domesticated independently, their long history of dual domestication and geographic spread has led to unintentional as well as intentional interspecific introgression, from pre‐colonial times to the modern era.^[^ ^49^, ^50^ ^]^ To identify ancient introgression segments (ancient‐ISs) of *G. hirsutum* that were introgressed from *G. barbadense* during its long‐term domestication and breeding history, published resequencing data from 250 *G. hirsutum* accessions and 22 *G. barbadense* accessions were collected for introgression analysis.^[^ ^26^, ^50^ ^]^ Among all 26 chromosomes, a total of 986 high‐quality introgression bins (MAF > 0.05) were identified, covering 211.04 Mb of the *G. hirsutum* genome. These introgression bins varied among chromosomes, from chromosome D04 (3 ancient‐IS bins, 0.83 Mb total) to A06 (260 ancient‐IS bins, 36.50 Mb total) (Figure S23 and Table S30, Supporting Information). The average length of ancient‐ISs was 0.21 Mb, 90% of which had lengths shorter than 0.4 Mb (Figure S24a, Supporting Information). Notably, most (97.26%) of the ancient‐ISs were identified in only a few (≈24%) accessions (Figure S24b, Supporting Information), indicating limited permeability in the accession pool.

**Figure 6:**
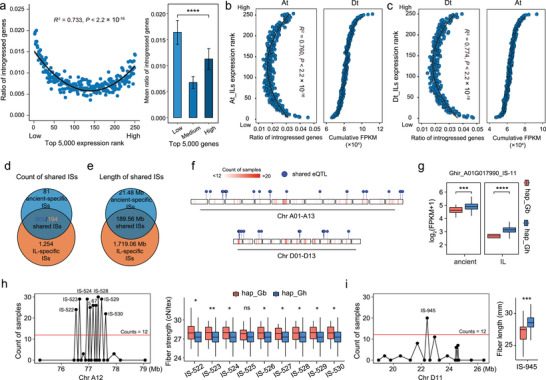
Characteristics of ancient introgression segments. a) The relationship between the expression rank and introgression segments. Left panel, dot plot showing the ratio of introgressed genes (y‐axis) at the corresponding expression rank (x‐axis). Right panel, bar plot showing the mean ratio of introgressed genes (y‐axis) at the corresponding expression rank (x‐axis). Low, 0%–5% of expression ranks; Medium, 25%–75% of expression ranks; High, 95%–100% of expression ranks. ****, *P* < 0.0001. Error bars are presented as mean ± SD. b) Relationship between expression rank and introgression segments from At. Left panel, dot plot showing the ratio of introgressed genes from At (x‐axis) at the corresponding expression rank (y‐axis). Right panel, dot plot showing the cumulative FPKM of introgressed genes from the Dt corresponding to homoeologous genes in At. c) Relationship between expression rank and introgression segments from Dt. Left panel, dot plot showing the ratio of introgressed genes from Dt (x‐axis) at the corresponding expression rank (y‐axis). Right panel, dot plot showing the cumulative FPKM of introgressed genes from At corresponding to homoeologous genes in Dt. d) Venn plot of counts of shared introgression segments between ancient‐ISs and IL‐ISs. e) Venn plot of the length of shared introgression segments between ancient‐ISs and IL‐ISs. f) Chromatin ideogram showing the distribution of ancient‐ISs and shared‐ISs between ancient‐ISs and IL‐ISs. Blue dot, shared eQTL. The heatmap from white to red represents more accessions with introgression segments. g) Box plots show a shared eQTL–eGene association between ancient‐ISs and IL‐ISs. Pink box, haplotype from *G. barbadense*; Blue box, haplotype from *G. hirsutum*. ***, *P* < 0.001; ****, *P* < 0.0001. Center line, median; box limits, first and third quartiles; whisker, 1.5× interquartile range. h) An example of FS‐related ISs. Left panel, dot plot for the location (x‐axis) and counts of accessions (y‐axis) with corresponding FS‐related ISs. Black dot, FS‐related IS. Red line, the threshold of accession counts when MAF < 0.05. Right panel, box plots showing the FS value between different haplotypes. Pink box, haplotype from *G. barbadense*; Blue box, haplotype from *G. hirsutum*. ns, *P* > 0.05; *, *P* < 0.05; **, *P* < 0.01. Center line, median; box limits, first and third quartiles; whisker, 1.5× interquartile range. i) An example of FL‐related ISs. Left panel, dot plot for the location (x‐axis) and counts of accessions (y‐axis) with corresponding FL‐related ISs. Black dot, FL‐related IS. Red line, the threshold of accession counts when MAF < 0.05. Right panel, box plots show FL values between different haplotypes. Pink box, haplotype from *G. barbadense*; Blue box, haplotype from *G. hirsutum*. ***, *P* < 0.001. Center line, median; box limits, first and third quartiles; whisker, 1.5× interquartile range.

We used 250 published transcriptome datasets of fiber samples at 15 DPA to calculate gene expression levels in *G. hirsutum* ^[^ ^26^ ^]^ for these ancient‐IS regions. Similar to introgression segments identified in the introgression line population, the ancient‐ISs were significantly associated with extreme expression of introgressed genes (R^2^ = 0.733, *P* < 2.2×10^−16^) (**Figure** **6**a; Figure S24c, Supporting Information). Additionally, ancient‐ISs from each subgenome also impacted the expression of homoeologous genes from the other subgenome (Figure 6b and c), in contrast to random gene pairs (Figure S24d and e, Supporting Information). These data support the above conclusions that genetic regulatory effects of introgression segments may include extreme expression of homoeologous genes in addition to older introgression segments.

To provide additional evidence for this idea, 13089 published eQTLs were collected to overlap with ancient‐ISs.^[^ ^26^ ^]^ We identified 827 ancient‐ISs involving eQTLs associated with 1762 genes (Table S30, Supporting Information). Among 986 ancient‐ISs, 91.69% (905 introgression bins, 189.56 Mb) overlapped with only 15.47% (194) of IL‐ISs (Figure 6d and e). It seems likely that ILs provided a novel resource for interspecies segment exchange. We found that 43 of 55 shared eQTL–eGene associations identified from 905 shared introgression segments had the same regulatory direction between the ancient group and the IL group (Figure 6f; Figure S24f, Supporting Information). For example, Ghir_A01G017990 (*ACC1*) encoded an acetyl‐CoA carboxylase, which is essential for very‐long‐chain fatty acid elongation, was regulated by the ancient‐IS IS‐11 (in IL, this segment was named M67) (Figure 6g). Accessions with segments from *G. hirsutum* exhibited higher expression levels than accessions with segments from *G. barbadense* in both the ancient group and IL group (Figure 6g). There were 25 ancient‐ISs associated with the expression of homoeologous genes (Table S30, Supporting Information), which was significantly higher than the numbers of ancient‐ISs associated with the expression of randomly selected gene pairs in a 1000‐permutation test (ancient‐ISs > 12, *P* < 0.01), indicating that extreme expression of homoeologous genes may be caused by the same eQTL.

To associate ancient‐ISs with fiber development, we integrated published GWAS QTLs with ancient‐ISs.^[^ ^26^, ^51^, ^52^, ^53^, ^54^ ^]^ We found that 15 and 6 ancient‐ISs were covered with FS QTLs and FL QTLs, respectively (Figure S23, Supporting Information), but only 8 and 5 ancient‐ISs were identified as FS‐related ISs and FL‐related ISs with haplotype analysis. For 8 FS‐related ISs (IS‐522 to IS‐524 and IS‐526 to IS‐530), the segments from *G. barbadense* may be favorable for breeding high‐FS cotton (Figure 6h). For 5 FL‐related ISs, for example, IS‐945, the segments from *G. hirsutum* may be favorable for breeding high‐FL cotton (Figure 6i). Although some ancient‐ISs have no significant effect on fiber quality improvement, these introgression sites may be related to other important aspects of cotton biology that were not investigated but which might be related to adaptation.^[^ ^8^, ^50^ ^]^

### 2.9 Validation of Fiber Length‐Related Candidate Genes

**Figure 7:**
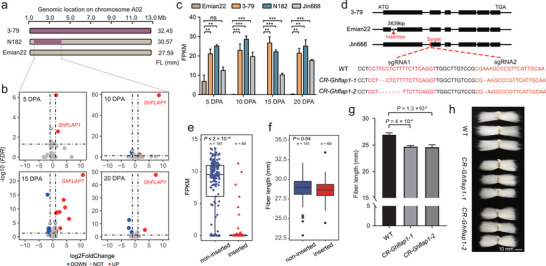
Functional analysis of *GhFLAP1*. a) Location of the introgression segment on chromosome A02 and fiber length of N182. The purple segment represents the introgression segment from 3–79. b) Volcano map of DEGs in the introgression segment of A02 chromosome in Emian22 and N182. Red circles represent genes that are up‐regulated, blue circles represent genes that are down‐regulated, gray circles represent genes that are not differentially expressed, and dashed lines represent thresholds for screening, with selection criteria of |log2FoldChange| > 1 and *FDR* < 0.05. c) Expression patterns of *GhFLAP1* of four cotton samples in four timepoints. Statistical significance was derived from Student's *t* test, as indicated by ns, *P* > 0.05; **, *P* < 0.01; ***, *P* < 0.001. Error bars are presented as mean ± SD. d) Structure of *GhFLAP1* in different materials and the null coding sequence of the *Ghflap1* mutants. Black box, exons; red triangle, inserted sequence; CR, CRISPR knockout lines. The red line indicates the guide RNA site. e and f) Expression level and fiber length of inserted and non‐inserted groups, with n representing the number of samples in the group. Center line, median; box limits, first and third quartiles; whisker, 1.5× interquartile range. Significance is tested by Wilcoxon sum rank test. g and h) Fiber length of the *Ghflap1* mutants (n = 3) and WT (n = 3). Statistical significance was derived from Student's *t* test. Error bars are presented as mean ± SD. Scale bars in (h), 10 mm.

To further explore the relevance of introgression to our understanding of functional genes associated with fiber quality, we focused on the introgression segment on chromosome A02 in introgression line N182, which displays the highest fiber length in the introgression line population (**Figure** **7**a). In this line, the expression of one gene (GhirA02G003720, named *GhFLAP1*) was significantly different at the four fiber timepoints between the parents Emian22 and N182 (Figure 7b). *GhFLAP1* encodes a fluctuating light acclimation protein (FLAP1) in *Arabidopsis*. The expression level of *GhFLAP1* in Emian22 was significantly lower than that in 3–79 and N182 (Student's *t* test, *P* < 0.01 and *P* < 0.001; Figure 7c), but there was no difference between 3–79 and N182 (Student's *t* test, *P* > 0.05; Figure 7c). A comparison of the coding region in Emian22 and 3–79 revealed that there was a 3,639 bp insertion in the first exon of *GhFLAP1* in Emian22, which led to the division of the first exon into two exons (Figure 7d; Figure S25, Supporting Information). Not surprisingly, this insertion led to the deletion of six amino acids in the translated protein of *GhFLAP1* in Emian22 (Figure S25, Supporting Information).

To explore the genetic variation of *GhFLAP1* more generally, we analyzed genotype and expression using resequencing data and RNA‐seq data from a *G. hirsutum* population described previously.^[^ ^26^ ^]^ The results showed that 250 accessions could be divided into two groups, among which 69 accessions (defined as the insertion group) had a sequence insertion in *GhFLAP1* and the remaining 181 accessions (defined as the non‐insertion group) did not. Both the expression level of *GhFLAP1* and fiber length in the non‐insertion group were much higher than those in the insertion group (Wilcoxon sum rank test, *P* ≤ 2 × 10^−16^ and *P* = 0.04; Figure 7e and f).

To characterize the gene function of *GhFLAP1*, we used CRISPR/Cas9‐mediated gene editing to knock out *GhFLAP1* in the accession Jin668, which does not have a sequence insertion in *GhFLAP1*, and the expression level was much higher than that in Emian22, which does have the insertion (Student's *t* test, *P* < 0.001; Figure 7c). We obtained two mutant lines, *CR‐Ghflap1‐1* and *CR‐Ghflap1‐2* (Figure 7d). Compared with the wild‐type (WT), the fiber length of both mutant lines decreased significantly (Student's *t* test, *P* = 4 × 10^−4^ and *P* = 1.3 × 10^−3^; Figure 7g and h; Figure S26a, Supporting Information). GO and KEGG enrichment analyses of Emian22 and N182 at 10 DPA showed that the upregulated genes were significantly enriched in the oligosaccharide metabolic and plant signal transduction pathways, containing 13 genes related to auxin biosynthesis, which may imply the involvement of *GhFLAP1* (Figures S26b,c and S27, Supporting Information). Moderate auxin can promote fiber elongation and positively correlate with fiber development, while high auxin concentration can inhibit growth.^[^ ^55^, ^56^, ^57^, ^58^, ^59^ ^]^ Taken together, our results show that *GhFLAP1* may be involved in fiber elongation, and the genotype from *G. barbadense* is favorable for fiber length improvement.

## 3 Discussion

It is well understood that gene expression is controlled by both *cis‐*regulatory sequences as well as *trans‐*acting factors, and that understanding these interactions can offer insights into developmental networks, phenotypes, and evolutionary transitions.^[^ ^4^, ^60^, ^61^, ^62^, ^63^, ^64^, ^65^ ^]^ Less well‐understood are the additional complexities introduced by allopolyploidy, where two divergent suites of *cis* and *trans* regulatory elements are united in a common nucleus, leading to both intra‐ and inter‐subgenomic interactions.^[^ ^47^, ^48^ ^]^ Here we analyzed the expression dynamics of introgressed genes from one allopolyploid species (*G. barbadense* 3–79) following their introgression into a second allopolyploid species (*G. hirsutum* Emian22). The experimental design was such that we could interrogate both *cis* and *trans* controls for both homoeologs of the introgressed genes, based on transcriptome data at four timepoints of fiber development. A regulatory model was established by comparing the expression changes of genes with their expression in the parents (Figure 1a).

Our results indicate that when the exogenous segment is introgressed as a *cis*‐regulatory element into the regulatory environment of the genome, genes may be expressed above or below the level in the native regulatory environment (Figure 1a). Although *cis*‐*trans* interactions may result in increased gene expression, decreased expression may be more common. For example, it has been shown that genes tend to be downregulated in the *Solanum pennellii* introgression region of the nightshade family.^[^ ^66^ ^]^ Our study also showed that genes in the introgression region had extreme expression, and this extreme expression was more likely to be low expression (Figure 4a and b; Figure S16, Supporting Information). Collectively, the present research demonstrates that gene introgression in allopolyploids sets in motion expression changes that may be transgressive or more extreme than parental expression levels, and also which are more complex than in diploids, often affecting homoeologous expression levels via presumed novel trans interactions.

An important consequence of the multiplicity of *cis* and *trans* interactions in polyploids is their effects on gene expression of duplicated (homoeologous) genes.^[^ ^47^, ^48^ ^]^ Our introgression lines permitted insights into factors that affect regulatory changes in homoeologous gene pairs following introgression of exogenous segments that include only one of the two homoeologs. The results showed that after one gene in a homologous gene pair is introgressed, the expression of the other non‐introgressed duplication will be biased (Figure 4c and d, Figure 6b and c; Figure S18, Supporting Information). The presence of shared eQTLs and introgressed copies as eQTLs of non‐introgressed copies is the main reason for the co‐variation of the expression levels of homoeologous gene pairs^[^ ^67^ ^]^ (Figure 4f; Figure S20 and Table S30, Supporting Information). The regulation of subgenomes in allopolyploids has been shown to correlate with the *cis*‐*trans* relationships of the parents of different origins.^[^ ^49^ ^]^ Shared eQTLs observed here suggest that both copies (At and Dt) from two different subgenomes are regulated in *trans* by the same sequence, or that the introgressed sequence is *cis*‐acting on one copy and *trans*‐acting on the other. After one homoeolog is replaced by an exogenous segment, the expression level of its homoeologous copy often is altered. For example, after one homoeolog GhirA06G010000 was introgressed, its expression level increased compared with that of Emian22, and the expression level of the non‐introgressed copy gene GhirD06G001950 also increased (Figure 4g). We speculate that divergence in TF binding or other *cis*‐regulatory sequences between the native and the exogenous sequence, or changes of TF concentrations, may alter expression levels of the introduced gene and/or its homoeolog, thus creating compensatory or antagonistic relationships.^[^ ^48^, ^68^, ^69^ ^]^

Because of the increased use of genomic data, introgression events are now understood to be widespread in organisms. Interspecific introgression in plants has been shown to play an important role in adaptation and phenotypic innovation.^[^ ^10^, ^11^, ^12^, ^13^, ^70^ ^]^ In this study, we identified introgression segments from *G. barbadense* and found significant associations with fiber quality‐related traits at some loci in 250 *G. hirsutum* accessions and 323 introgression lines (Figure 5a; Figure S23, Supporting Information). Comparing the introgression positions and genetic regulatory of ancient‐ISs and IL‐ISs, we found that most ancient introgression sites and regulatory relationships overlapped with introgression lines (Figure S23 and Table S30, Supporting Information). We found that 43 shared eQTL have been utilized in cultivated *G. hirsutum* accessions, and accounting for less than 1% of the identified eQTLs in introgression lines (Figure 6f; Figure S24f, Supporting Information). This result indicates that some of the regulatory loci in *G. barbadense* have already been applied in modern cultivars and implies the potential space for the regulatory relationship identified in ILs to improve cotton fiber quality in the future.

In the cases of both ancient group and the IL group, we observed that introgression regions were not uniformly distributed across the reference genome, with certain regions being more or less susceptible to introgression than others^[^ ^71^ ^]^ (Figures S6 and S23, Supporting Information). The number of introgression sites in modern populations is significantly higher for recent than for ancient introgression sites, possibly due to natural selection in the latter, where introgression segments were eliminated following hybridization due to fitness disadvantages or hybrid incompatibility, which is well‐known in cotton and previously was called F2 breakdown resulting from “cryptic structural differentiation”.^[^ ^72^, ^73^ ^]^ In cases of modern introgression, however, selection may have led to retention of genomic segments associated with excellent crop phenotypes, and thus become fixed by artificial selection.

In summary, our study provides a wealth of information on the relationships among genomic segments and fiber quality, which can serve as a reference for cotton fiber breeding and accelerate the development of new cotton varieties. The analysis of regulatory perturbation effects of introgression segments provides an opportunity for the exploration of how gene introgression affects phenotypic variation in cotton, and likely in other species as well.

## 4 Experimental Section

### Plant Materials

In a previous study, it was constructed introgression lines to introduce chromosome segments from sea‐island cotton accession 3–79 (*Gossypium barbadense*) into upland cotton accession Emian22 (*Gossypium hirsutum*).^[^ ^35^, ^41^ ^]^ Emian22, the recipient parent, has high yield but medium fiber quality. The donor parent 3–79 was the standard line in genetics and cytogenetics for *G. barbadense*, with excellent fiber quality. To further clarify the regulatory differences in fiber development between *G. hirsutum* and *G. barbadense*, Emian22, 3–79, and 323 different ILs were cultivated in Huanggang, China, in 2020. Fresh young leaves of Emian22 were collected four weeks after growth and immediately frozen in liquid nitrogen for genomic DNA extraction. Cotton bolls of all plants were collected at 5 DPA, 10 DPA, 15 DPA, and 20 DPA, immediately frozen in liquid nitrogen and stored at −80 °C for total RNA extraction. Emian22, 3–79 and N182 (IL with excellent fiber quality) were represented by three biological replicates, each collected from at least three cotton plants.

The T_0_ of the *Ghflap1* mutants were planted in a greenhouse in Wuhan, China. T_1_ and Jin668 were cultivated in a cotton experimental field of Huazhong Agricultural University for phenotypic identification and functional characterization. Similarly, cotton fibers from Jin668 were collected at four timepoints of development according to the above method for total RNA extraction, which also included three biological replicates.

### Genome Sequencing and Assembly

Sequencing reads were collected using Nanopore and Illumina. A *de novo* DNA library of Emian22 was constructed following the standard manufacturer (Oxford Nanopore Technologies) protocol for Nanopore genome sequencing. The final library was sequenced on the Oxford Nanopore Technologies platform. Clean data were assembled by SMARTdenovo (https://github.com/ruanjue/smartdenovo), and then the next‐generation DNA‐seq data were used to perform three rounds of correction using Pilon^[^ ^74^ ^]^ software. Finally, a total of 238.43 Gb of Nanopore sequences, with a genome coverage depth of 120×, were generated to assemble the genome into 453 contigs (Tables S1–S4, Supporting Information), guided by the k‐mer genome survey analysis (Figure S1, Supporting Information). The draft contig was divided into fragments with a length of 50 kb, and the polished contigs of Emian22 were clustered by LACHESIS^[^ ^75^ ^]^ and anchored onto 26 pseudochromosomes. BUSCO^[^ ^76^ ^]^ (v 5.1.3) and LTR Assembly Index^[^ ^77^ ^]^ (LAI) were used to assess the completeness of the genome assemblies.

### Comparative Genomic Analysis of Emian22 and 3–79/TM‐1

The genomes of Emian22 and 3–79/TM‐1 were compared using MUMmer^[^ ^78^ ^]^ (v 4.0.0) with the following parameters: ‐mum ‐c 90 ‐l 40. The specific alignment sequences in the two genomes were preserved by running parameter −1. The show‐snp command was used to identify SNPs and Indels in both genomes. We used ANNOVAR^[^ ^79^ ^]^ to annotate the effects of SNPs and InDels. SNP and InDel densities were calculated by using the sliding window method (windows: 1 Mb; steps: 200 kb). SNP density between Emian22 and 3–79 was 7.24 per kilobase (Kb), and the InDel density was 1.34 per Kb (Figure 1b; Tables S12 and S13, Supporting Information). The SNP frequency of the A subgenome was slightly higher than that of the D subgenome (Figure S2, Supporting Information), consistent with the observation between *G. hirsutum* TM‐1 and 3–79.^[^ ^41^ ^]^ We observed ≈11 808 genes with changes in protein sequences or structures (Figure 1c; Tables S12 and S13, Supporting Information). Large alterations included stop codon gain (5521 SNPs and 1121 InDels), stop codon loss (1140 SNPs and 194 InDels), start codon loss (433 SNPs and 78 InDels), and open reading frame shifts (15993 InDels) (Figure 1c; Figure S3, Tables S12 and S13, Supporting Information).

Identification of inversions and translocations was accomplished by filtering through two sets of parameter settings in MUMmer software. Two sets of parameter settings were used: ‐i 90 −1 ‐r ‐q and ‐i 90 ‐g ‐r –q, where −1 represents 1‐to‐1 matching between two genomes and the ‐g parameter indicates that there can be no rearrangement in the comparison region between genomes. Nonallelic regions between genomes were obtained by excluding allelic regions after 1‐to‐1 matching. These nonallelic regions were defined as inversions or translocations.^[^ ^80^ ^]^

We selected regions that were not aligned between genomes using “show‐diff” in MUMmer. These unaligned regions were reserved as potential unique regions of Emian22 and 3–79/TM‐1 and then compared with the respective genomes using blastn. The final unique regions of Emian22 and 3–79/TM‐1 met the conditions of coverage >50% and identity >90%.

The SyRI^[^ ^81^ ^]^ (v 1.6) comparison tool was used to identify structural variants (SVs) between the Emian22 and 3–79/TM‐1 genomes using minimap2^[^ ^82^ ^]^ (v 2.17) with ‐ax asm5 ‐eqx. SVs were divided into 3 types: PAVs, inversions, and translocations. The final tabulation of inversions, translocations, and PAVs preserves the overlapping positions from MUMmer and SyRI software.

### Identification of Introgression Segments

DNA resequencing data^[^ ^35^, ^41^ ^]^ from the published introgression line population were mapped to the reference genome Emian22 using BWA software.^[^ ^83^ ^]^ Uniquely mapping reads were used to identify SNPs using Sentieon.^[^ ^84^ ^]^ Subsequently, SNPs were filtered with following parameters: ′QUAL < 30.0 || QD < 2.0 || FS > 60.0 ||MQ < 40.0 || SOR > 4.0″ with adjacent SNPs separated by at least 10 bp.

The sliding‐window method was used to identify introgression segments from *G. barbadense* to *G. hirsutum* in each IL.^[^ ^41^ ^]^ The Emian22 genome was divided into 541285 bins (total SNPs 16238562 between parents), and each bin consisted of 30 consecutive SNPs. Using the allele frequency (*G. barbadense/G. hirsutum*), if the ratio in each bin was greater than 25/5, it was regarded as a homozygous genotype of *G. barbadense*, and if it was less than 5/25, it was considered a homozygous genotype of *G. hirsutum;* between these two extremes, the segment was considered a heterozygous genotype.

### RNA Sequencing and Analysis

For each introgression line and 4 cotton materials (Emian22, 3–79, N182 and Jin668), cotton fibers were separated from the ovule and ground into powder in liquid nitrogen. Total RNA was extracted using a Polysaccharide Polyphenol Plant Total RNA Extraction Kit (TIANGEN, DP441). Sequencing libraries were prepared using the Hieff NGS Ultima Dual‐mode mRNA Library Prep Kit for MGI (YEASEN, 13330ES96). Each library was sequenced on the MGISEQ‐2000 platform by paired‐end 150 bp sequencing.

High‐quality clean RNA‐seq data from ILs were mapped to the reference genome of Emian22/3‐79^[^ ^41^ ^]^ using HISAT2^[^ ^85^ ^]^ (v 2.2.0). RNA‐seq data from four cotton materials, each with three biological replicates, were mapped to the reference genome of Emian22. SAMtools^[^ ^86^ ^]^ (v 1.9) was used to sort sequencing reads and filter out those that represented PCR duplication and with a mapping quality less than 30. The gene expression levels (FPKM) of all the samples was generated using StringTie^[^ ^87^ ^]^ (v 2.1.4).

### Expression Analysis of *G. barbadense*‐Specific Genes in Introgression Lines

The position of introgression segments in 3–79 were reconstructed based on the principle of genome synteny. It was extracted 150 bp upstream and 150 bp downstream from the breakpoints of each introgression segment in Emian22. The segment with a length of 300 bp were mapped to 3–79 genome using blastn (e‐value < 1e‐5), and the segments were retained with identity > 90% and coverage > 50%. According to the location of the breakpoints for each introgression segment, we determined the position of introgression segments in 3–79 (Table S22, Supporting Information).

The genes located within the introgression segments were compared with gene predictions in 3–79. The genes that were retained were designated as species‐specific genes in 3–79 by filtering out genes located in collinear regions in Emian22 and 3–79 species. Differentially expressed genes were defined as those with a change of at least 3 folds in expression level and exhibiting differential expression in at least 2/3 of introgression lines.

### Defining Stable and Altered Patterns

Differentially expressed genes between the introgression lines and the parents in the population were defined as those where the fold change was greater than or equal to 3. To explore the expression level of introgressed genes after introgression, the expression level of introgressed genes with those of the two parents was compared. When there was no difference in expression between the introgression line and the parent 3–79, these genes were defined as stably expressed, or “stable”. Genes with different expression levels between the introgression line and the parent 3–79 were defined as belonging to the “altered” pattern. To improve confidence of stable and altered genes, it was filtered them based on their expression types in the corresponding non‐introgressed lines. According to the definitions of stable and altered genes, stable genes exhibited differential expression in at most 7 samples (1/4 of the maximum number of samples with introgression segments introgressed) in the corresponding non‐introgressed lines, while altered genes showed differential expression in at least 20 samples (3/4 of the maximum number of samples with introgression segments introgressed). The term “number of samples with introgression segments introgressed” refers to the frequency of the same introgression position occurring in the population (Table S24, Supporting Information). Ultimately, highly confident stable was retained and altered genes for subsequent GO (Gene Ontology) and KEGG (Kyoto Encyclopedia of Genes and Genomes) enrichment analyses using TBtools.^[^ ^88^ ^]^

### Genetic Model

The multi‐locus model for quantitative traits in ILs is as follows:

(1) $$y_{i}=\;\mu +\sum_{j\;=\;1}^{m}w_{ij}a_{j}+\sum_{j\;=\;1}^{m}v_{ij}d_{j}+\varepsilon_{i}$$

where *y_i_* is the *i*th phenotypic observation value of quantitative trait (i=1,2,…,n); μ is the total average; *a_j_* and *d_j_* are the additive and dominance effects of the *j*th locus, respectively; and *w_ij_* and *v_ij_* are their corresponding indicator variables. We assumed that alleles A and a of each locus are derived from the donor and background parents, so:

(2) $$w_{ij}=\left\{\begin{array}{cc} 1 & \mathrm{for}\;\;AA\\ 0 & \mathrm{for}\;\;Aa\\ -1\; & \mathrm{for}\;\;aa \end{array}\right.\;\mathrm{and}\;v_{ij}=\left\{\begin{array}{cc} 0 & \mathrm{for}\;\;AA\\ 1 & \mathrm{for}\;\;Aa\\ 0 & \mathrm{for}\;\;aa \end{array}\right.$$

ε_*i*_ is the residual error.

### Parameter Estimation

The LASSO^[^ ^89^ ^]^ was a popular method for linear regression using L_1_ penalty to achieve the sparse solution. For a given value of λ, a nonnegative complexity parameter, LASSO solves the following problem.

(3) $$\min_{\mu ,a_{j},d_{j}{|}_{j\;=\;1}^{m}}\left(\frac{1}{2n}\sum_{i\;=\;1}^{n}{\left(y_{i}-\mu -w_{ij}a_{j}-v_{ij}d_{j}\right)}^{2}+\lambda \sum_{j\;=\;1}^{m}\left(\left|a_{j}\right|+\left|d_{j}\right|\right)\right)$$

As λ decreases, the number of QTLs of nonzero‐effect increases. We used the *glmnet* package to estimate all the additive and dominance effects, it applies coordinate descent to solve the problem^[^ ^90^ ^]^ (https://cran.r‐project.org/web/packages/glmnet/).

### Hypothesis Test

After estimating all additive and dominance effects of all loci, LOD scores for all nonzero loci were calculated using the likelihood ratio test. The numbers of lines with genotypes AA, Aa and aa at the *k*th locus were *n*_1_, *n*_2_, and *n*_3_ respectively. Under the null hypothesis *H*_0_:  *a_k_* =  0, $y_{i}^{k}=y_{i}\;-\mu -\sum_{j\neq k}w_{ij}a_{j}-\sum_{j\;=\;1}^{m}v_{ij}d_{j}$ followed a normal distribution for all three genotypes,^[^ ^39^ ^]^ and the natural logarithm likelihood function was indicated by:

(4) $$L_{0}=\sum_{i=1}^{n}\ln f\left(y_{i}^{k}|\mu_{0},\sigma_{0}^{2}\right)$$

where $f(y_{i}^{k}|\mu_{0},\sigma_{0}^{2})$ is the density function of normal distribution $N(\mu_{0},\sigma_{0}^{2})$, and μ_0_ and $\sigma_{0}^{2}$ were estimated using $\mu_{0}=\sum_{i=1}^{n}y_{i}^{k}/n$ and $\sigma_{0}^{2}=\sum_{i=1}^{n}{\left(y_{i}^{k}-\mu_{0}\right)}^{2}/n$, respectively. Under alternative hypothesis *H_A_*: *a_k_* ≠ 0, the natural logarithm likelihood function was indicated by:

(5) $$\begin{array}{ccc} L_{A} & = & \sum_{i=1}^{n_{1}}\ln f\left(y_{i}^{k}|\mu_{1},\sigma^{2}\right)\;+\sum_{i\;=n_{1}\;+1}^{n_{1}+n_{2}}\ln f\left(y_{i}^{k}|\mu_{2},\sigma^{2}\right)\\ & & +\sum_{i\;=n_{1}\;+n_{2}+1}^{n}\ln f\left(y_{i}^{k}|\mu_{3},\sigma^{2}\right) \end{array}$$

where $\mu_{1}=\sum_{i=1}^{n_{1}}y_{i}^{k}/n_{1}$, $\mu_{2}=\sum_{i=n_{1}+1}^{n_{1}+n_{2}}y_{i}^{k}/n_{2}$, $\mu_{3}=\sum_{i=n_{1}+n_{2}+1}^{n}y_{i}^{k}/n_{3}$ and $\sigma^{2}=\left(\sum_{i=1}^{n_{1}}{\left(y_{i}^{k}-\mu_{1}\right)}^{2}+\sum_{i=n_{1}+1}^{n_{1}+n_{2}}{\left(y_{i}^{k}-\mu_{2}\right)}^{2}+\sum_{i=n_{1}+n_{2}+1}^{n}{\left(y_{i}^{k}-\mu_{3}\right)}^{2}\right)/n$.

Thus, the LOD score of likelihood ratio test for an additive effect was:

(6) $$LOD\;=\frac{-2\left(L_{0}-L_{A}\right)}{2\ln10}$$

The LOD score for dominance effect was similarly calculated. In this study, the LOD threshold of significant QTL was set to ≥ 2.5. Meanwhile, the LOD threshold of significant eQTL was set to ≥ 4. In the eQTL mapping, we used the R base function *qqnorm* to transform the gene expression level as the quantitative trait phenotypic value. The methods developed in this study have been uploaded to GitHub, and the repository can be accessed via the following link: https://github.com/liguocat/cssl.

### Monte Carlo Simulation Studies

In Monte Carlo simulation studies, there were 1448 segments in 307 cotton ILs (introgression lines containing introgression segments). Eight QTLs with various degrees of dominance (*d*/*a*, from −3 to + ∞) were simulated, including one additive, one dominance, and six additive‐dominance QTLs. Here, the genetic variance for each QTL was set as 1 so that the sum of all the QTL variances was eight, the residual error variance was set as 2, and the total average (μ) was set as 100. Thus, the phenotypic value of each IL was simulated via $y_{i}=\;\mu +\sum_{j\;=\;1}^{8}w_{ij}a_{j}+\sum_{j\;=\;1}^{8}v_{ij}d_{j}+\varepsilon_{i}$, where $\varepsilon_{i}∼N\left(0,2\right)$ is residual error and the other symbols are the same as those in model (1). The number of replicates was 1000.

### Identification of eQTL Hotspots and eQTL Sharing and Specificity

The average number of eGenes regulated by each eQTL in the population (17.9 eGenes/eQTL) was calculated, and then we defined eQTLs whose eGenes regulated by an eQTL reached 5‐fold as eQTL hotspots.

The effect size distribution of eQTLs was observed, and the effect values were between −2 and 2. Therefore, the additive effect was used for analysis, and an effect size within 2 times between two periods was indicative of eQTL sharing. Otherwise, the eQTL was defined as a specific eQTL.

### Network Construction of Genetic Perturbation in ILs

Based on the eQTL information of the four timepoints, some eQTLs using the corresponding differentially expressed genes in each introgression line were filtered. Each eQTL‐eGene association in the network must ensure that the eGene regulated by the introgression segment was a differentially expressed gene in the corresponding ILs. A total of 12497 eQTL‐eGene associations were retained.

### Expression Analysis of Homoeologous and Random Gene Pairs

Homoeologous gene pairs in the Emian22 genome were identified, and filtered out homoeologous gene pairs that were introgressed in the same sample and those that were not introgressed in either sample. Introgressed gene frequencies in the expression rankings were calculated according to the reported method^[^ ^43^, ^46^ ^]^ for the introgressed copies of the preserved homoeologous gene pairs. For each gene, the normalized gene expression measurements of all 323 introgression lines were sorted into 323 ranks. Subsequently, the number of genes introgressed in this rank is counted. The number of introgressed gene counts for these expression ranks are then summed for all genes and plotted from lowest to highest rank. If extreme expression occurs in introgression copies, a quadratic regression is fitted to the curve. The same method to calculate the gene expression of non‐introgressed copies of all genes in the order of expression rank of introgression copies (from low to high) was used. The gene ID of the introgressed copy in the random gene pair was the same as the gene ID of the introgressed copy of its homoeologous gene pair. The total expression of random genes for non‐introgressed copies was calculated using the same method as that for homoeologous genes for non‐introgressed copies.

### Co‐Localization of QTLs and eQTLs

To assess the probability that molecular traits represented by eQTLs have the same causal variation as phenotypic traits represented by QTLs, colocalization analysis was performed. For each QTL, the interval with a distance of 300 kb from the significant QTL (LOD ≥ 2.5) was included in the candidate range. For eQTL results, the list of markers within 1 Mb from candidate genes for analysis was extracted. When the candidate regions of a QTL and an eQTL met the significance criteria in the same site, this indicated that the QTL and eQTL were colocalized.

### Identification and Analysis of Ancient Introgression Segments

To identify ancient introgression segments (ancient‐ISs) into *G. hirsutum* from *G. barbadense*, published resequencing data of 250 *G. hirsutum* accessions^[^ ^26^ ^]^ and 22 *G. barbadense* accessions^[^ ^50^ ^]^ were downloaded and mapped to the reference genome of *G. hirsutum*.^[^ ^41^ ^]^ The strategy of mapping reads and calling SNPs was described in our previous study.^[^ ^26^ ^]^

A published simplified workflow to identify ancient‐ISs between 250 *G. hirsutum* accessions and 22 *G. barbadense* accessions was followed.^[^ ^8^ ^]^ In short, the frequency difference (Fre_Gh_ – Fre_Gb_) of the genotype between two populations was calculated SNP‐to‐SNP, and 500 adjacent SNPs were clustered as a bin to calculate the average frequency difference. The bins with an average frequency difference > 0.2 and MAF > 0.05 were identified as ancient‐ISs.

To annotate ancient‐ISs, the regions of published GWAS QTLs and eQTLs were lifted to the same reference genome of *G. hirsutum* ^36^ by LiftOver^[^ ^91^ ^]^ (https://genome.sph.umich.edu/wiki/LiftOver). Ancient‐ISs overlapping with published GWAS QTL or eQTL regions were identified as trait‐ or expression‐associated ancient‐ISs.

### CRISPR/Cas9‐Mediated Gene Editing

The CRISPR/Cas9 knockout construct for *GhFLAP1* was designed to produce defined deletions in the second exon using sgRNA. Then, the plasmid vector was introduced into the cotton recipient cultivar Jin668 by Agrobacterium‐mediated genetic transformation. Next, the Hi‐TOM technique (https://www.x‐mol.com/groups/Wang_kejian/hitom) was used to construct a high‐throughput sequencing library to detect the base change of *GhFLAP1* in the mutant, and the primers used are summarized in the Table S31 (Supporting Information). Sequencing data were uploaded to the Hi‐TOM online analysis website (http://www.hi‐tom.net/hi‐tom/) to analyze the mutant sequence and corresponding genotype information of the *Ghflap1* mutants.

### Identification and Analysis of Insertion Sequence

In the previous study, *G. hirsutum* accessions were resequenced to construct genomic variation maps.^[^ ^92^ ^]^ Subsequently, 251 of these accessions were planted, and cotton fibers were collected at 15 DPA for RNA‐seq to analyze the regulatory mechanism of fiber development.^[^ ^26^ ^]^ A total of 250 upland cotton accessions (excluding 3–79) were selected for analysis to determine the presence of insertion sequence in populations. First, 50 kb upstream and downstream of *GhFLAP1* in the Emian22 genome were used as the reference genome, and Sentieon was used to map the resequencing data to the reference genome. Sequencing reads were sorted using SAMtools, and duplicate reads generated during PCR amplification and those with a mapping quality less than 30 were filtered out. BEDTools^[^ ^93^ ^]^ was used to convert the mapped bam file into a bed file representing the location in the genome. Finally, the bed file was subjected to counting. If there were reads covering 50 bp before and after the breakpoint of the insertion sequence, the *GhFLAP1* locus was considered to have an insertion sequence; otherwise, we inferred the absence of an insertion sequence.

## Conflict of Interest

The authors declare no conflict of interest.

## Author Contributions

X.C., X.H., and G.L. contributed equally to this work. M.W. and X.Z. designed the experiments and managed the project. Z. Lin developed the introgression line population. X.C., X.H., J.Y., R.W., X.L., Y.P., M.Z., Yuqi Zhang and S.L. collected fiber samples and performed experiments. X.C. performed genome assembly and annotation. X.C., J.Y., C.E.G., Z. Liu and Z.Q. performed genome re‐sequencing and RNA‐Seq data analysis. G.L. and Yuan‐ming Zhang developed the method for QTL and eQTL identification. M.W. and X.C. proposed a framework of introgression‐mediated genetic perturbations. J.Y. performed ancient‐IS analysis. X.H. performed functional characterization of candidate genes. X.C., X.H. and J.Y. wrote the manuscript draft, and M.W., X.Z., C.E.G. and J.F.W. interpreted experimental results and participated in the writing and revision of the manuscript.

## Supporting information

Supporting Information

Supplemental Dataset 1

## Data Availability

All raw sequencing data generated in this paper have been deposited into the National Center for Biotechnology Information database (BioProject ID: PRJNA970991). The genome assembly and annotation are available at the Figshare website (https://figshare.com/projects/G_hirsutum_Emian22_genome/166916). The raw sequencing data for the genome assembly are available in the Sequence Read Archive (SRA) under accession numbers SRR29873798 to SRR29873801. The SRA numbers of DNA‐seq data and RNA‐seq data of introgression lines are provided in Table S21 (Supporting Information).
