# Supplementary material for: Genetic Regulatory Perturbation of Gene Expression Impacted by Genomic Introgression in Fiber Development of Allotetraploid Cotton
Source: Adv Sci (Weinh). 2024 Aug 28;11(40):2401549. doi: 10.1002/advs.202401549 (PMC11515910; doi:10.1002/advs.202401549)
Supplement: Supplementary file 1 — Supporting Information [file ADVS-11-2401549-s001.pdf]

ADVANCED SCIENCE
Open Access

Supporting Information

for *Adv. Sci.*, DOI 10.1002/advs.202401549

Genetic Regulatory Perturbation of Gene Expression Impacted by Genomic Introgression in Fiber Development of Allotetraploid Cotton

Xinyuan Chen, Xiubao Hu, Guo Li, Corrinne E. Grover, Jiaqi You, Ruipeng Wang, Zhenping Liu, Zhengyang Qi, Xuanxuan Luo, Yabin Peng, Mengmeng Zhu, Yuqi Zhang, Sifan Lu, Yuan-ming Zhang, Zhongxu Lin, Jonathan F. Wendel, Xianlong Zhang* and Maojun Wang**

Supplemental Notes

Assessment of genome size in Emian22

Based on Illumina platform generated three 350 bp Emian22 genome DNA libraries, after filtering, a total sequencing depth of approximately 52× and a total of 122.43 Gb of high-quality data were obtained. Basic genome characteristics such as genome size, repeat sequences, and heterozygosity were assessed based on k-mer distribution (k = 21). When k = 21, an average k-mer depth around 44, corresponding to the main peak on the k-mer distribution graph, was observed (**Figure S1, Supporting Information**). Sequences with k-mer depths appearing more than twice the depth of the main peak, i.e., depths greater than 89, were classified as repeat sequences. Sequences with k-mer depths appearing at half the depth of the main peak, i.e., depths around 22, were classified as heterozygous sequences. Following two hypotheses that all k-mer distributions from sequenced reads cover the entire genome and that k-mer frequencies along the depth gradient follow a Poisson distribution, the genome size (G) was defined as G = k-mer number/k-mer depth, where the k-mer number is the total number of k-mers, and k-mer depth is the frequency occurring more frequently than other frequencies. The total number of k-mers obtained from sequencing data was 106,082,863,743, and after filtering, 103,557,307,944 k-mers were used for genome length estimation, resulting in an estimated genome size of approximately 2.3 Gb. Based on the k-mer distribution analysis, the estimated content of repeat sequences is approximately 66.56%, with no distinct peaks indicating heterozygosity. The estimated heterozygosity is about 0.13%. Consequently, the Emian22 genome is characterized as highly repetitive and complex due to its large size.

Identification of centromeres in Emian22

In plants, active centromeres are enriched with the centromere specific histone H3 variant CENH3. We collated previously published CENH3 ChIP-Seq (chromatin immunoprecipitation sequencing) data[1] and mapped it to the reference genome using

the BWA-MEM[2] algorithm, producing BAM files. Subsequently, we employed SAMtools[3] to filter out reads with base quality scores below 20 and retained uniquely aligned reads with at most one base mismatch. CENH3 ChIP-Seq peaks were identified using SICER2[4], revealing potential centromeric regions. For the identification of CENH3 domains, we utilized 200 bp windows, demanded a fold change of ≥5 over control, set a false-discovery rate (FDR) threshold of less than 0.01, and accepted gaps of up to 400 bp. By analyzing CENH3 ChIP-seq data, we detected 25 potential centromeric regions in the Emian22 genome, excluding chromosome D08 (**Table S8, Supporting Information**).

Validation of SVs in Emian22

Structural variations (SV) were validated through reads mapping of breakpoints, Hi-C heatmaps, and PCR experiment.

A total of 250 Gb of Nanopore reads was utilized to map the inversion breakpoints in the Emian22 genome. Minimap2[5] (v2.17) was employed to extract and align the 20 Kb flanking sequences of inversion breakpoints between TM-1 and Emian22. At the inversion breakpoints in Emian22, over 98.0% of sequences were spanned by more than 50 reads. Similarly, translocation events were screened using the same method, revealing that approximately 97.9% of fragments in Emian22 met the criterion of having more than 50 reads spanning the breakpoints. Consequently, 58.7 Mb of inversion fragments and 36.9 Mb of translocation fragments were identified.

Additionally, to confirm the authenticity of these structural variations, inversion events on chromosomes A06 and A08 were validated using Hi-C heatmaps and PCR experiment. Hi-C heatmaps were utilized to identify an inversion event at chromosome A08 in upland cotton (**Figure S5a, Supporting Information**), as previously documented in the literature[6]. For the inversion event on chromosome A06, PCR primers were designed to amplify the complete inversion fragments in Emian22 and TM-1 (**Figure S5b, Supporting Information**), and the authenticity of the fragments

was verified via Sanger sequencing.

Comparison of genome assembly in upland cotton

We collected published upland cotton genome data[1, 6-10] and conducted comparisons with Emian22. The comparative analysis reveals minimal differences in genome size between Emian22 and other upland cotton versions, with Emian22 exhibiting advantageous quantities of contigs and longer contig N50 lengths among existing genome assemblies (**Table S9, Supporting Information**). Additionally, we assessed the completeness of the genome, finding nearly all versions to achieve approximately 100% completeness, affirming the high quality of upland cotton genome assembly (**Table S9, Supporting Information**). It is worth noting that the Emian22 genome assembled in this study is being released for the first time, providing a crucial foundation for enriching the genetic resources of upland cotton.

Validation of introgression segments

According to the identified coordinates of the introgression segments, combined with variation information of Emian22 and 3-79 reference genomes, the regions within the introgression segments with PAV in the sequence of 3-79 and Emian22 were selected for verification. The primers were designed at both ends for PCR amplification, and the authenticity of the PCR results was confirmed by Sanger sequencing. We randomly selected 5 introgression lines for verification (**Figure S7, Supporting Information**). According to the agarose gel electrophoresis, PCR amplification using the DNA of introgression line and 3-79 as the template showed no difference in bands, while there were differences with Emian22, indicating that the introgression segment came from 3-79. It is worth noting that N210 and N75 did not amplify bands in Emian22 due to the low similarity of the flanking sequences of PAV, but the consistency with 3-79 bands can also be indicative. In order to ensure the authenticity of the experimental results, we performed Sanger sequencing on three of the amplification products, and the

sequencing results showed that the PCR products amplified using the introgression line and 3-79 as the template were consistent in sequence.

Comparative analysis of introgression segments

A total of 323 introgression lines were selected in this study, which included 9 single chromosome segment substitution lines (SSSLs) and 146 multiple chromosome segment substitution lines (CSSLs), compared with the study by Wang et al.[10] (including 168 SSSLs). To further evaluate the effects of population size and genome version on the identification of introgression segments, we analyzed the exogenous fragments from *G. barbadense* 3-79 within each introgression line in the two studies.

The identified introgression segments from Wang et al. study were mapped onto the Emian22 genome, revealing that 330 segments (derived from 156 introgression lines) overlapped with those identified in this research. The overlapping lengths of these segments amounted to 84.95% (overlapping lengths/total introgression lengths from 168 lines: 2.08/2.45 Gb), covering approximately 61.74% of the Emian22 genome (1.38/2.23 Gb). Compared to Wang et al.'s research, which covered about 65.21% of the TM-1 genome (1.46/2.23 Gb), no significant changes were observed. Although we noted that about 85% of the introgression segments overlapped, some introgression segments were not mapped to the Emian22 reference genome, suggesting that differences in genome versions could affect the identification of introgression segments. This underscores the importance of precise assembly of the Emian22 genome in identifying sequence variations in introgression population.

In this study, we identified 621 introgression segments among the newly added 155 introgression lines, totaling 2.36 Gb, which represent approximately 50.66% of the total length of all introgression segments (introgression lengths of 155 lines/total introgression lengths from 323 lines: 2.36/4.66 Gb). This highlights the significant contribution of the new lines to enhancing the value and quality of the research. We also compared the coverage of identified introgression segments within the Emian22

genome between existing and newly added lines, which were 64.18% (1.43/2.23 Gb) and 58.52% (1.31/2.23 Gb), respectively. By merging the data from both sets of lines, we observed an overall genome coverage of approximately 85.43% (1.91/2.23 Gb) (**Table S19, Supporting Information**), demonstrating that the additional introgression lines have laid the foundation for constructing a comprehensive gene expression regulatory network in this study. Furthermore, we analyzed the genomic coverage across each chromosome (**Figure S8, Supporting Information**), further validating the effectiveness of the new introgression lines in enriching the population resources.

Phytohormone measurement

About 0.1g of cotton fiber was ground in liquid nitrogen and three biological replicates were set for each sample. The sample was added with 80% methanol and placed under a 4℃ shaker overnight for two consecutive times. Indole-3-acetic-2,2-d2 acid (IAA; Sigma-Aldrich) and ^{2}H6-abscisic acid (ABA; Olchemim) is used as the internal standard in extraction. Quantitative determination of IAA and ABA was performed using the ABI 4000 Q-Trap system.

Supplemental Figures

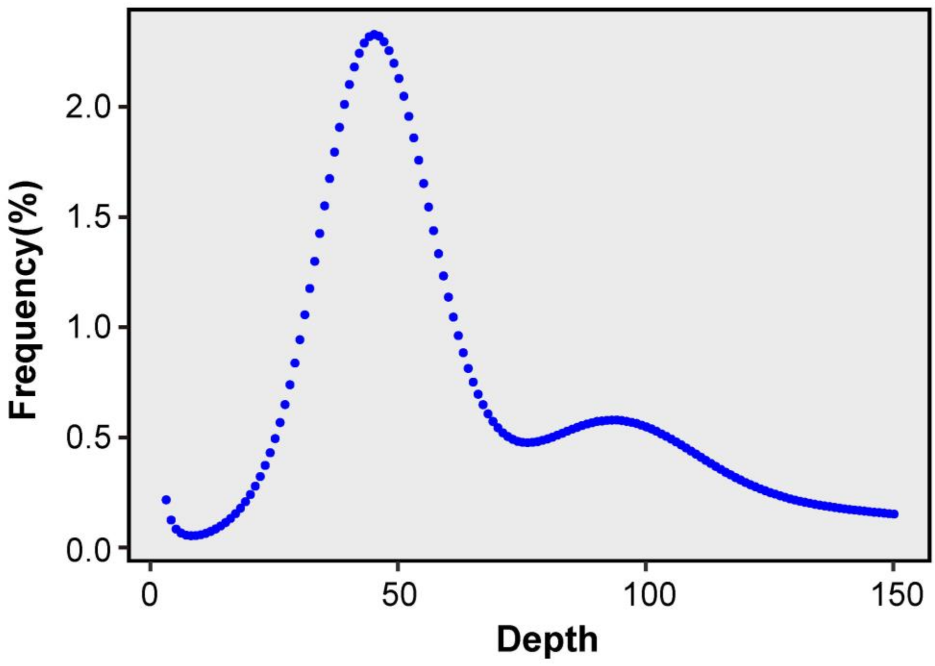

Figure S1 K-mer frequency distribution.

When k-mer = 21, a frequency peak value at 44 is observed and used to estimate the genome size.

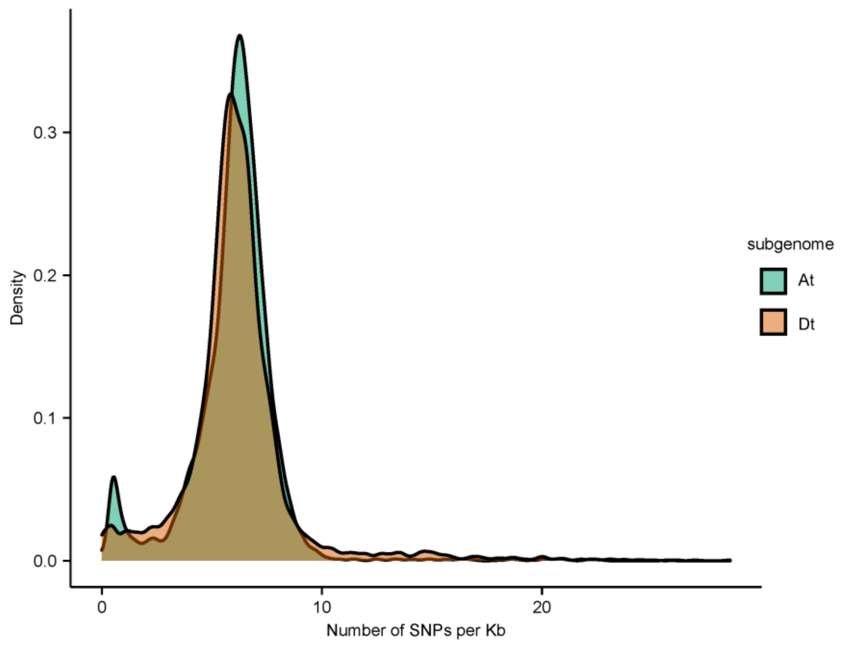

Figure S2 Density distribution of SNPs in the A and D subgenomes.

In this analysis, each chromosome was split into 1-Mb windows sliding 200-Kb. SNPs in each window were counted. The x-axis shows the number of SNPs in each 1-Kb size. The green area indicates SNP density distribution in the A-subgenome and yellow area indicates SNP density distribution in the D-subgenome.

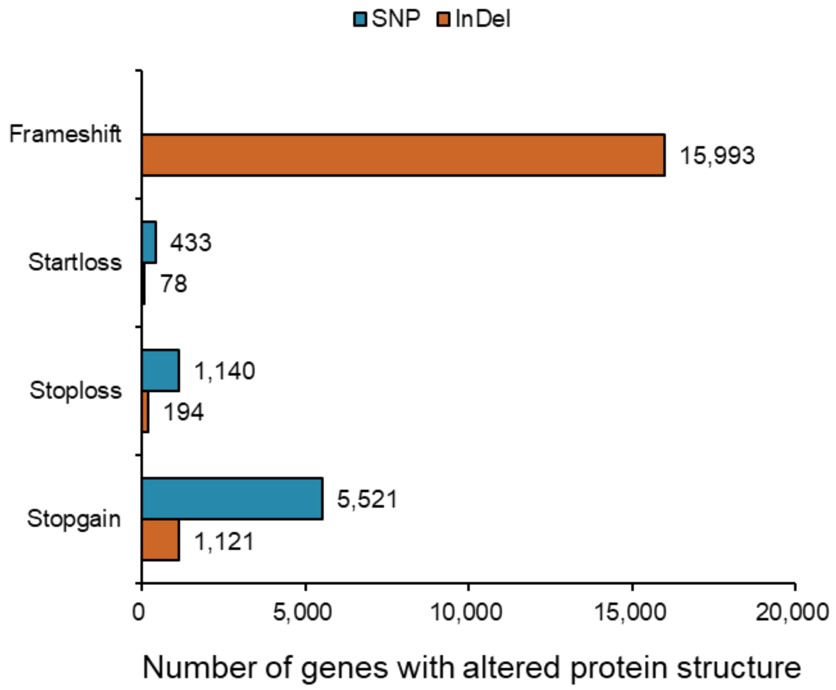

Figure S3 Distribution of the number of genes affected by genomic variation.

In Emian22, the number of genes with large changes in protein structure caused by SNPs and InDels. The x-axis represents the number of genes affected, and the y-axis shows 4 types of functional variation with large influence. Blue indicates that the type is from SNP, and orange indicates that the type is from InDel.

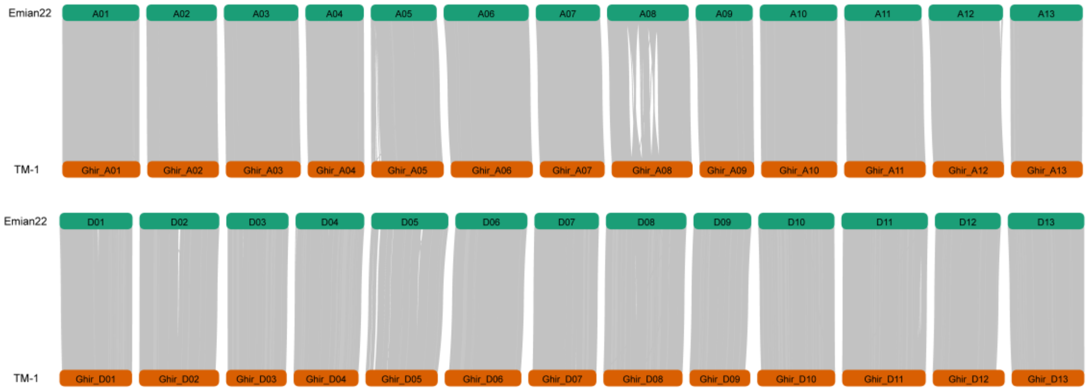

Figure S4 Genomic alignment of Emian22 and TM-1.

Alignment blocks for each chromosome are depicted by gray lines. The upper panel represents alignment information for the A subgenome, while the lower panel represents alignment information for the D subgenome.

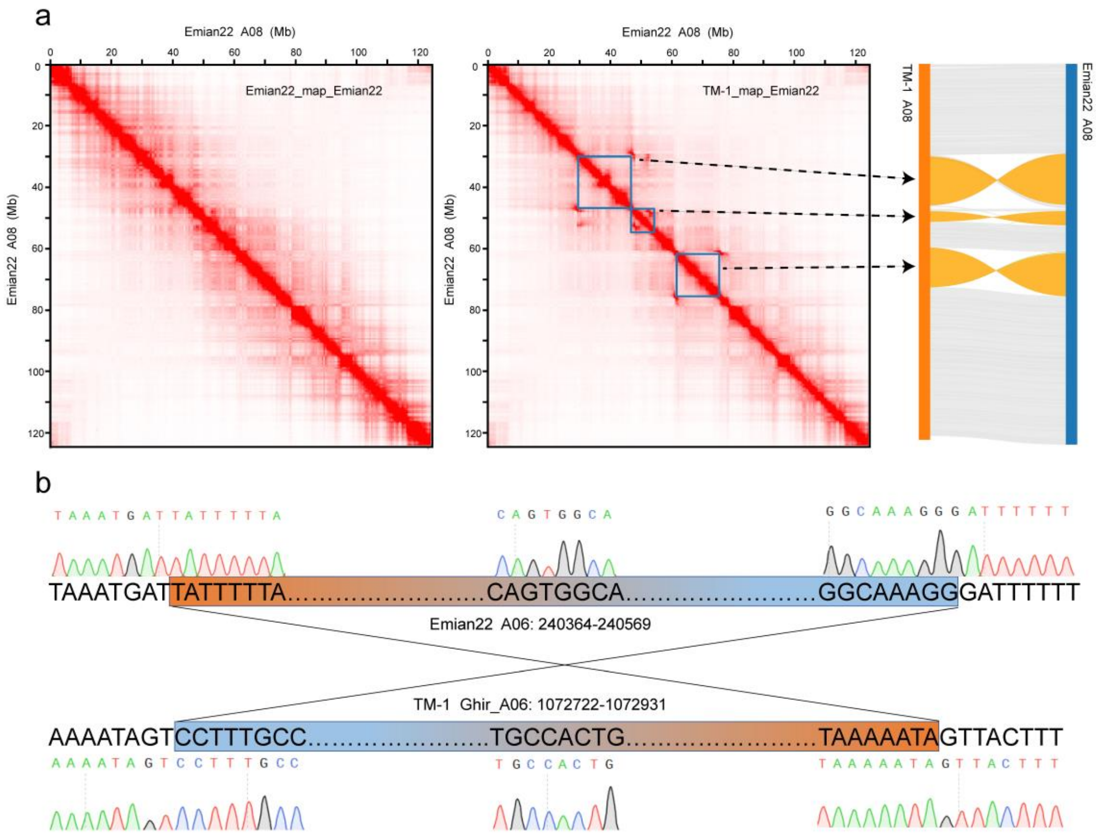

Figure S5 Validation of SVs in Emian22.

a, Large-scale inversions (SV1, SV2, and SV3) on A08 between Emian22 and TM-1. **b**, The sequence in the gradient box is inversion sequence, and the peak map is the result of Sanger sequencing.

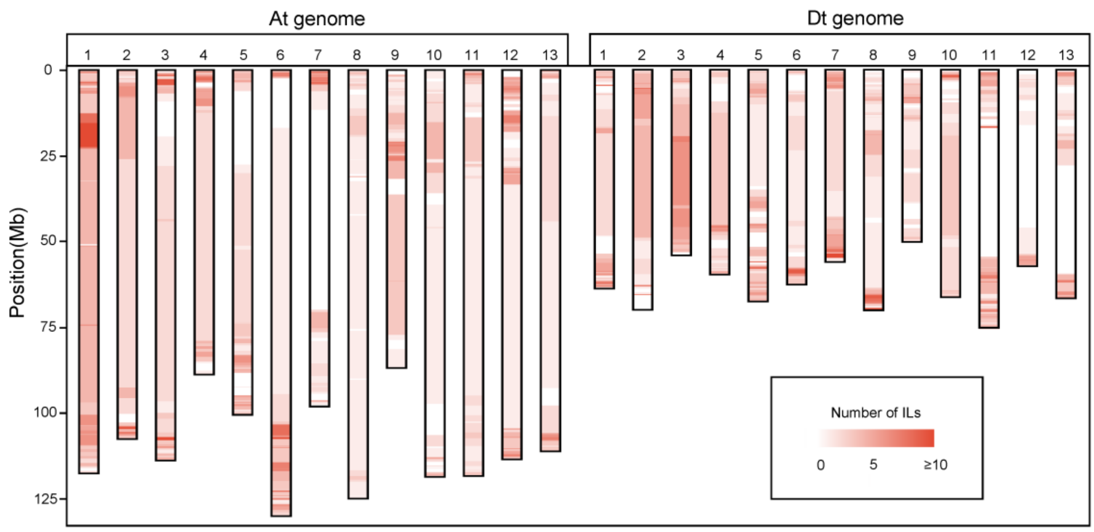

Figure S6 Distribution of introgression segments in the Emian22 genome.

The heatmap shows the number of introgression lines at each location. The x-axis represents the chromosomes in Emian22, and the y-axis represents the positions on each chromosome.

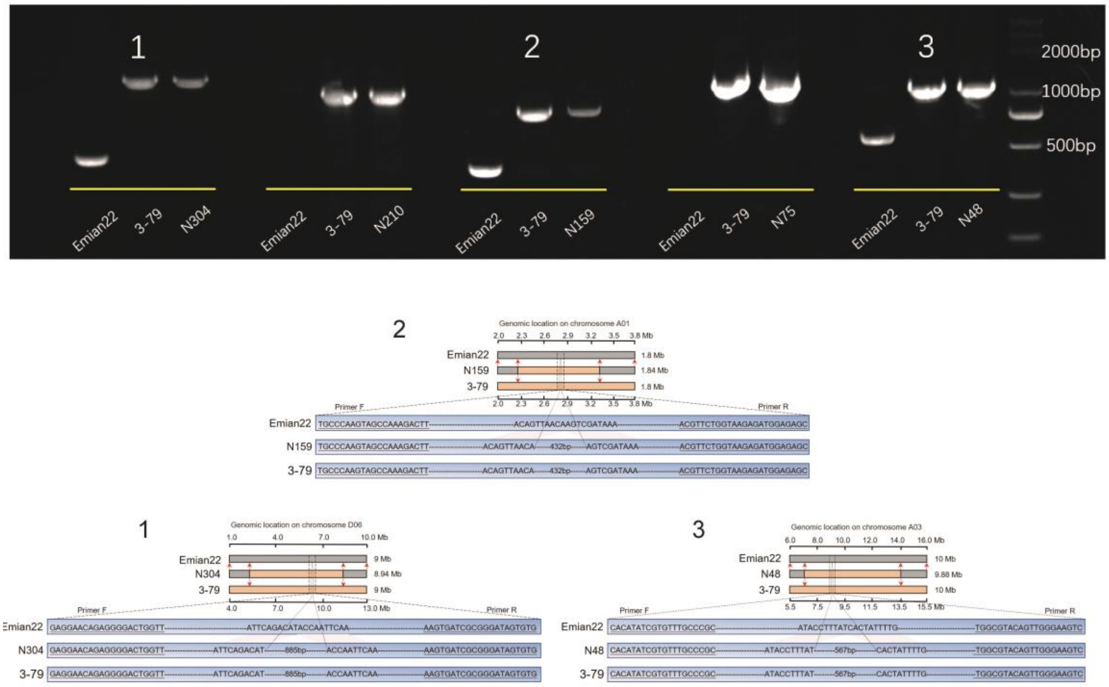

Figure S7 Validation of introgression segments.

Agarose gel electrophoresis and Sanger sequencing. The top number corresponds with the bottom number. The dotted line indicates the position and size of the PAV between Emian22 and 3-79. The dashed line represents the location of primers. The red arrow represents the source of chromosome fragments in introgression lines.

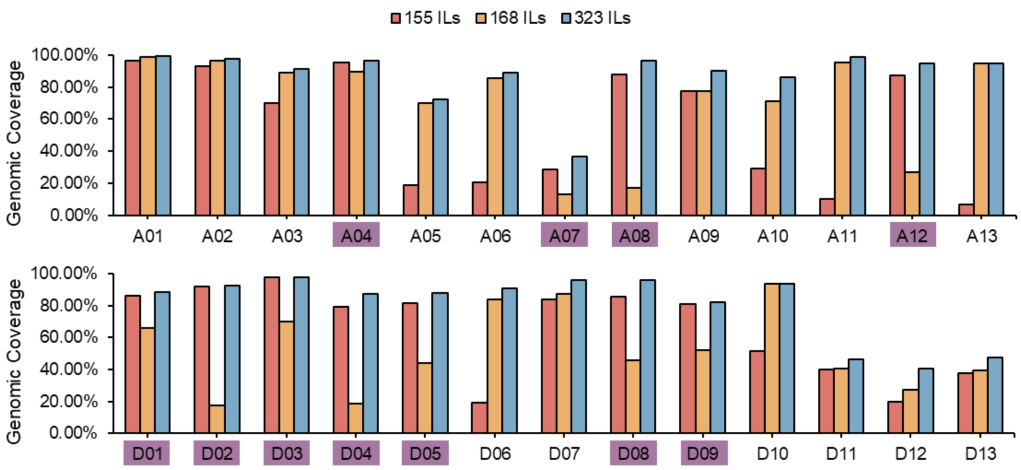

Figure S8 The coverage of each chromosome in the Emian22 genome among introgression lines from different sources.

The chromosomes highlighted in purple represent an increased genomic coverage in the newly added introgression lines compared to the introgression lines previously examined by Wang et al. (2019)[10].

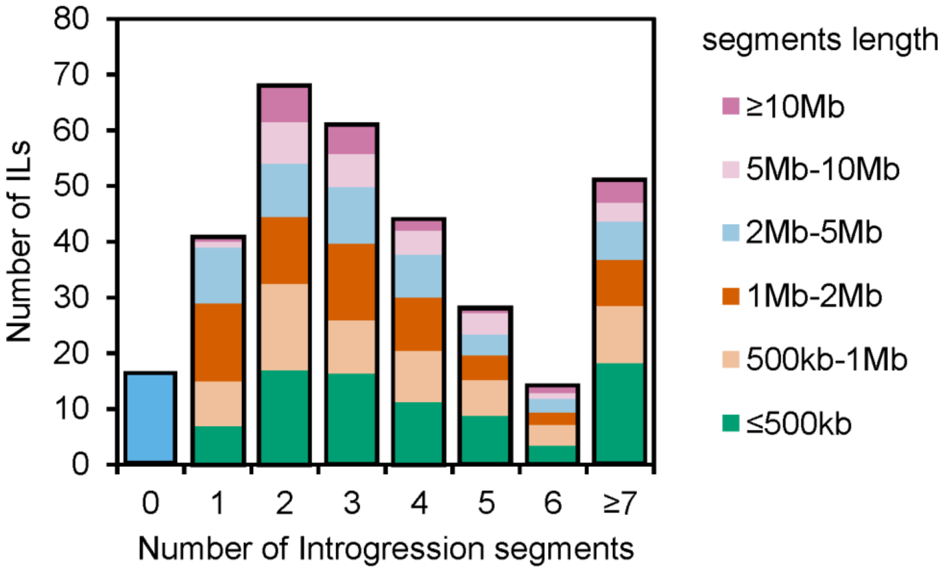

Figure S9 Distribution of length and number of introgression segments in introgression lines.

In the bar chart, the different colors in each column represent the length of introgression segments. The blue column represents the absence of introgression segments. The x-axis represents the number of introgression segments. The y-axis represents the number of introgression lines (IL).

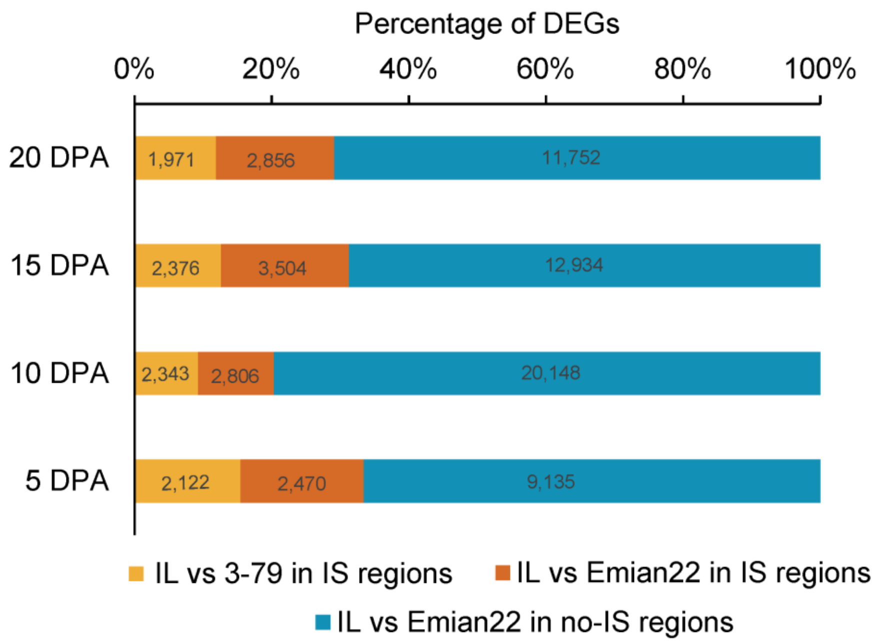

Figure S10 Number of differentially expressed genes between introgression lines and parents.

In this analysis, differentially expressed genes were defined as genes with an expression fold greater than 3 between the introgression lines and the parents. IS represents the genes in the introgressed regions, and no-IS represents the genes in the non-introgressed regions.

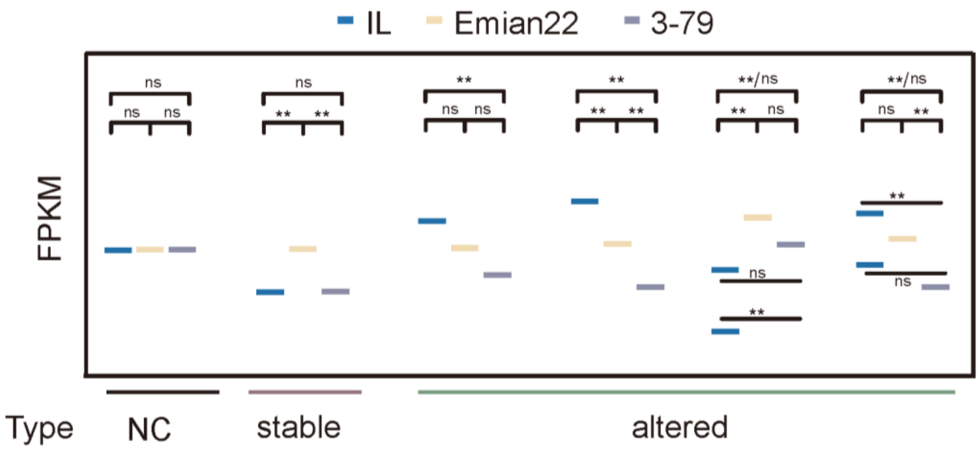

Figure S11 Definition of types of introgression genes.

The types of introgression genes demarcated in **Fig. 2d** are depicted. Blue indicates the expression level of genes in introgression lines, orange indicates the expression level of genes in Emian22, and gray indicates the expression level of genes in 3-79. Differentially expressed genes were defined as a fold change in expression level greater than or equal to 3.

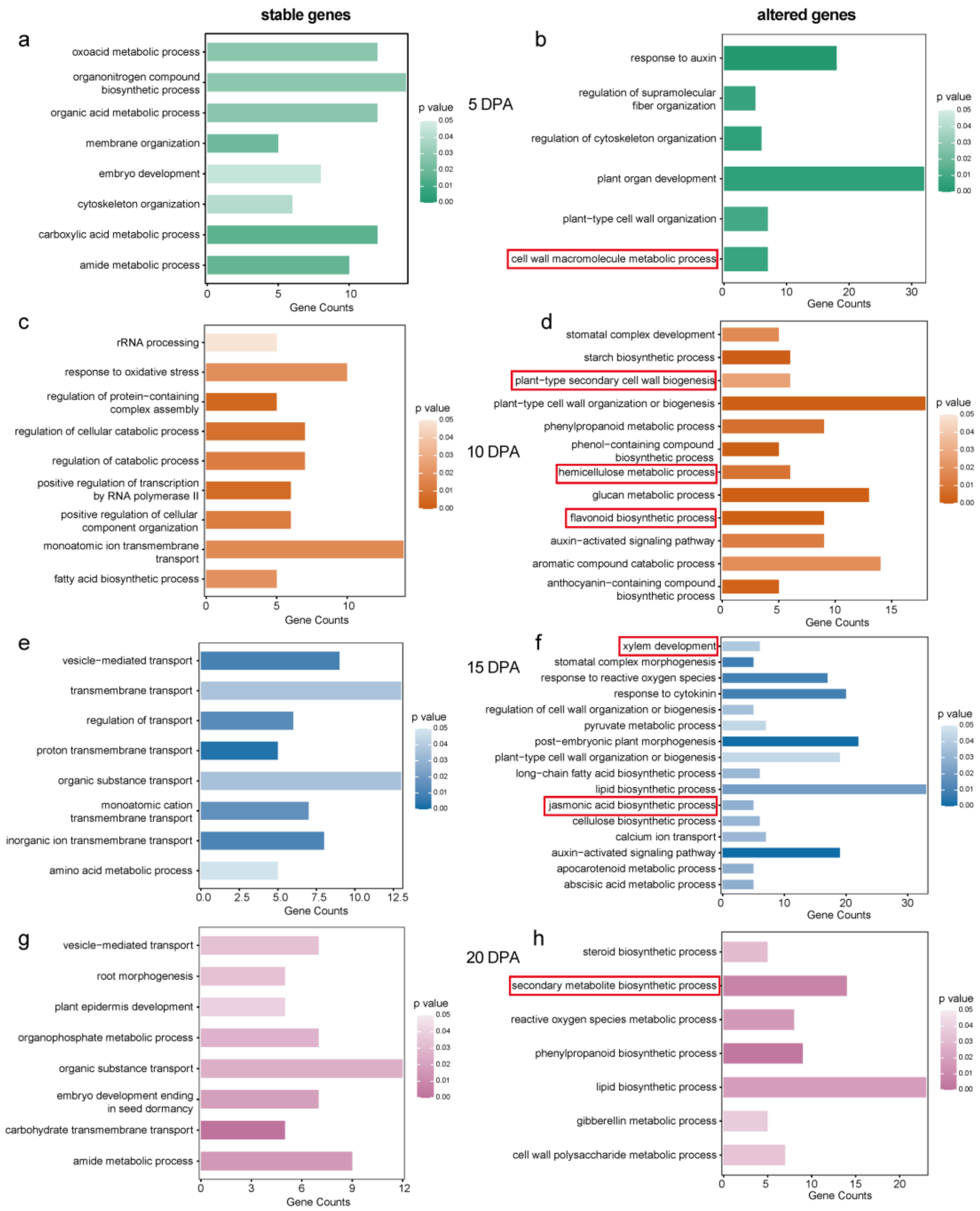

Figure S12 GO enrichment analysis of genes in considered “stable” or “altered” at four timepoints.

a, **c**, **e**, and **g** represent the enrichment pathways of stable genes in the four surveyed timepoints, respectively. **b**, **d**, **f**, and **h** represent the enrichment pathways of altered genes in the four timepoints, respectively. In the figure, green represents 5 DPA, orange represents 10 DPA, blue represents 15 DPA, and purple represents 20 DPA. All GO enrichment pathways in the figure show the terms related to Biological Process.

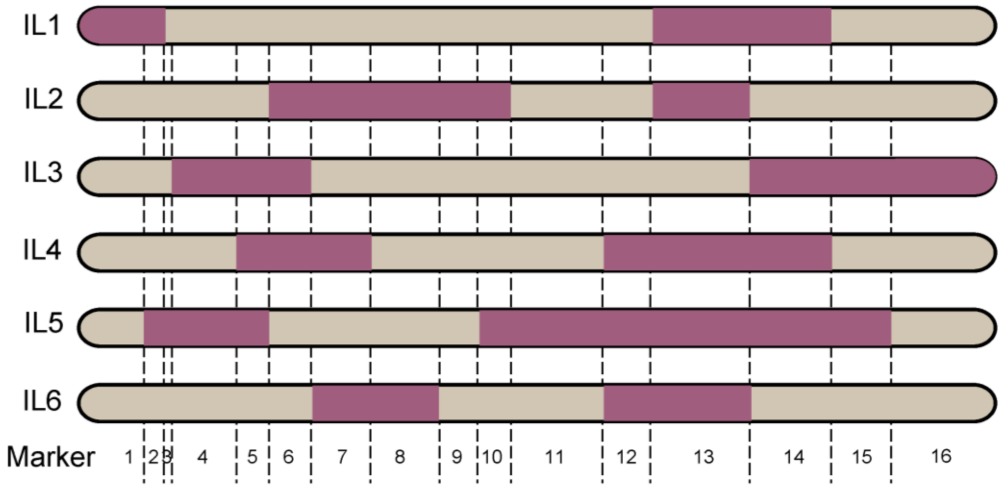

Figure S13 The relationship between markers and introgression segments.

Principles for defining markers. The brown color represents the chromosome of the Emian22 genome, and the dark purple represents the 3-79 introgression segments introgressed into the Emian22 genome. The positions of introgression segments for each introgression line are summarized and arranged in order of increasing position for each chromosome. Subsequently, the smallest overlapping segments in each introgression line are divided into a specific marker. The division of markers is based on the breakpoints of introgression segments.

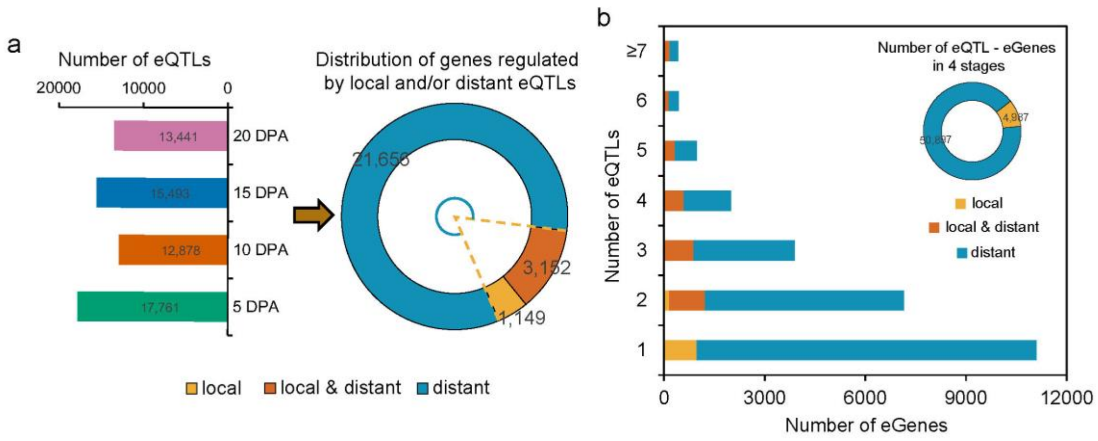

Figure S14 Identification of eQTLs using RNA-seq data in 4 timepoints.

a, The bar chart shows a summary of the number of eQTLs in 4 timepoints. In the figure, green represents 5 DPA, orange represents 10 DPA, blue represents 15 DPA, and purple represents 20 DPA. The donut plot shows the distribution of eGenes regulated by local and/or distal eQTLs. Yellow indicates genes regulated by local eQTLs. Orange indicates genes regulated by local and distal eQTLs. Blue indicates genes regulated by distal eQTLs.

b, Distribution of all eGenes in 4 timepoints. The ring graph represents the total number of eQTL-eGene associations identified in 4 timepoints. Yellow indicates local eQTLs. Blue indicates distal eQTLs. The bar graph shows the number of eGenes regulated by multiple eQTLs. Yellow indicates genes regulated by local eQTLs. Orange indicates genes regulated by local and distal eQTLs. Blue indicates genes regulated by distal eQTLs.

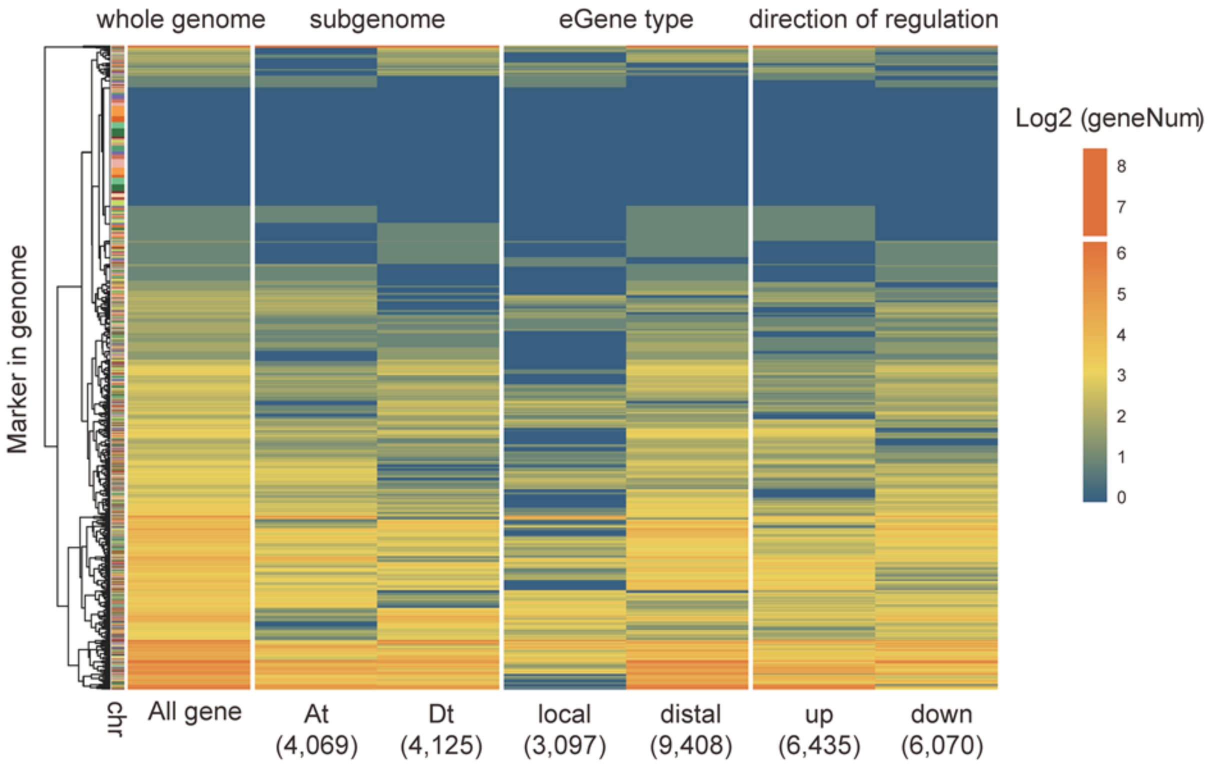

Figure S15 Genotype analysis in regulatory interference networks of introgression segments

Heatmap showing the distribution of the number of genes regulated by markers in the subgenome, eGene type and the direction of regulation. Chr represents the chromosome where each marker is located, and the chromosome color is the same as that in **Figure 3g**.

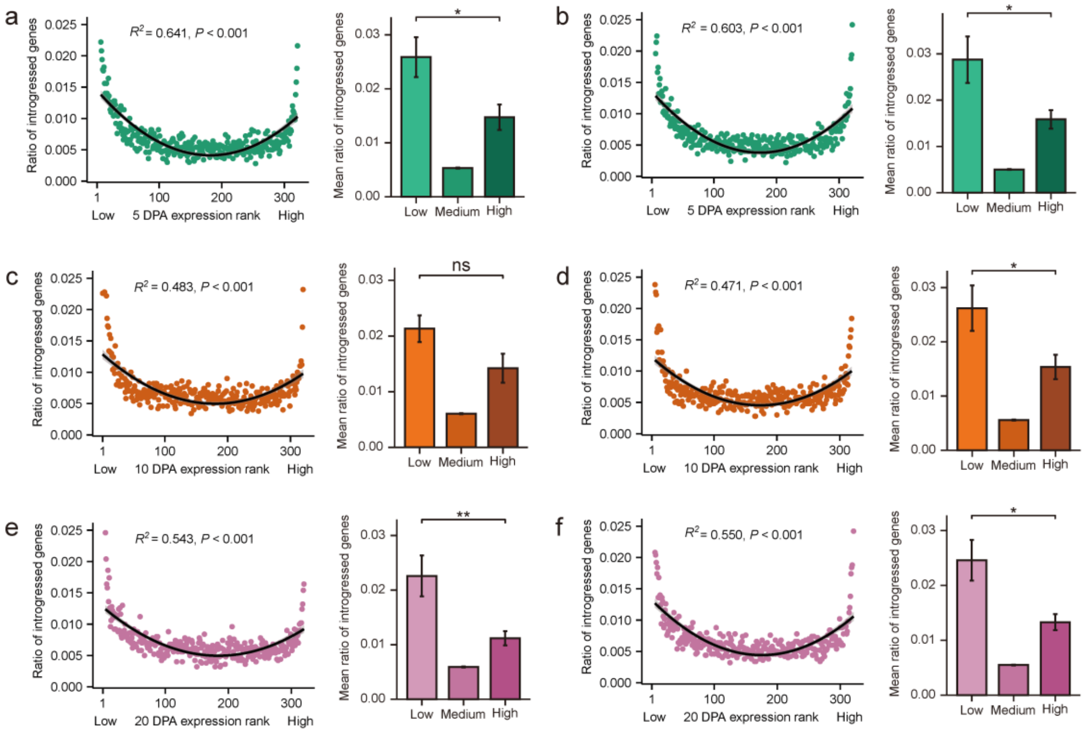

Figure S16 Extreme expression of introgression genes in IL population.

The panels on the left of **a**, **c**, and **e** show significant quadratic relationship between the expression rank of each line, in each of the top 5,000 most-expressed genes and the ratio of introgressed genes at 5 DPA, 10 DPA, and 20 DPA. The panels on the right of **a**, **c**, and **e** show comparison of mean ratio of introgressed genes for individuals in the bottom expression ranks (n = 16) versus the middle two quartiles (n = 161) versus the top expression ranks (n = 16) within the top 5,000 most-expressed genes.

The panels on the left of **b**, **d**, and **f** show significant quadratic relationship between the expression rank of each line, in each of the next 5,000 most-expressed genes and the ratio of introgressed genes at 5 DPA, 10 DPA and 20 DPA. The panels on the right of **b**, **d**, and **f** show comparison of mean ratio of introgressed genes for individuals in the bottom expression ranks (n = 16) versus the middle two quartiles (n = 161) versus the top expression ranks (n = 16) within the next 5,000 most-expressed genes.

All significances are tested by two-tailed Student's t test. ns, $P > 0.05$; *, $P < 0.05$; **, $P < 0.01$. Error bars are presented as mean ± SE.

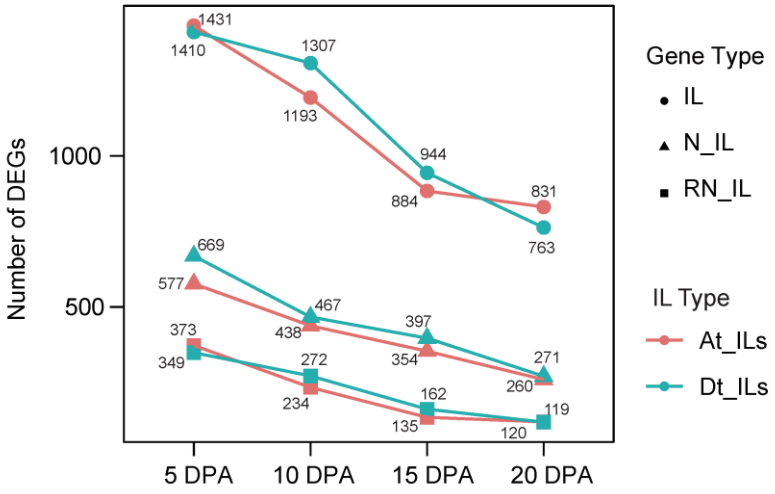

Figure S17 Number of differentially expressed genes between homoeologous gene pairs and parents.

In this analysis, we screened for homoeologous gene pairs that were introgressed in at least 3 samples and analyzed genes that differed between the homoeologous gene pairs and the parents. In At_ILs and Dt_ILs, the number of differentially expressed genes in the introgressed copy, non-introgressed copy, and random non-introgressed copy and parents are summarized, respectively. Differentially expressed genes here refers to a gene that is introgressed in multiple samples and has a significant difference in the expression level of the parent ($P < 0.05$). IL: homoeologous genes in introgressed regions; N_IL: homoeologous genes in non-introgressed regions; RN_IL: random genes in non-introgressed regions.

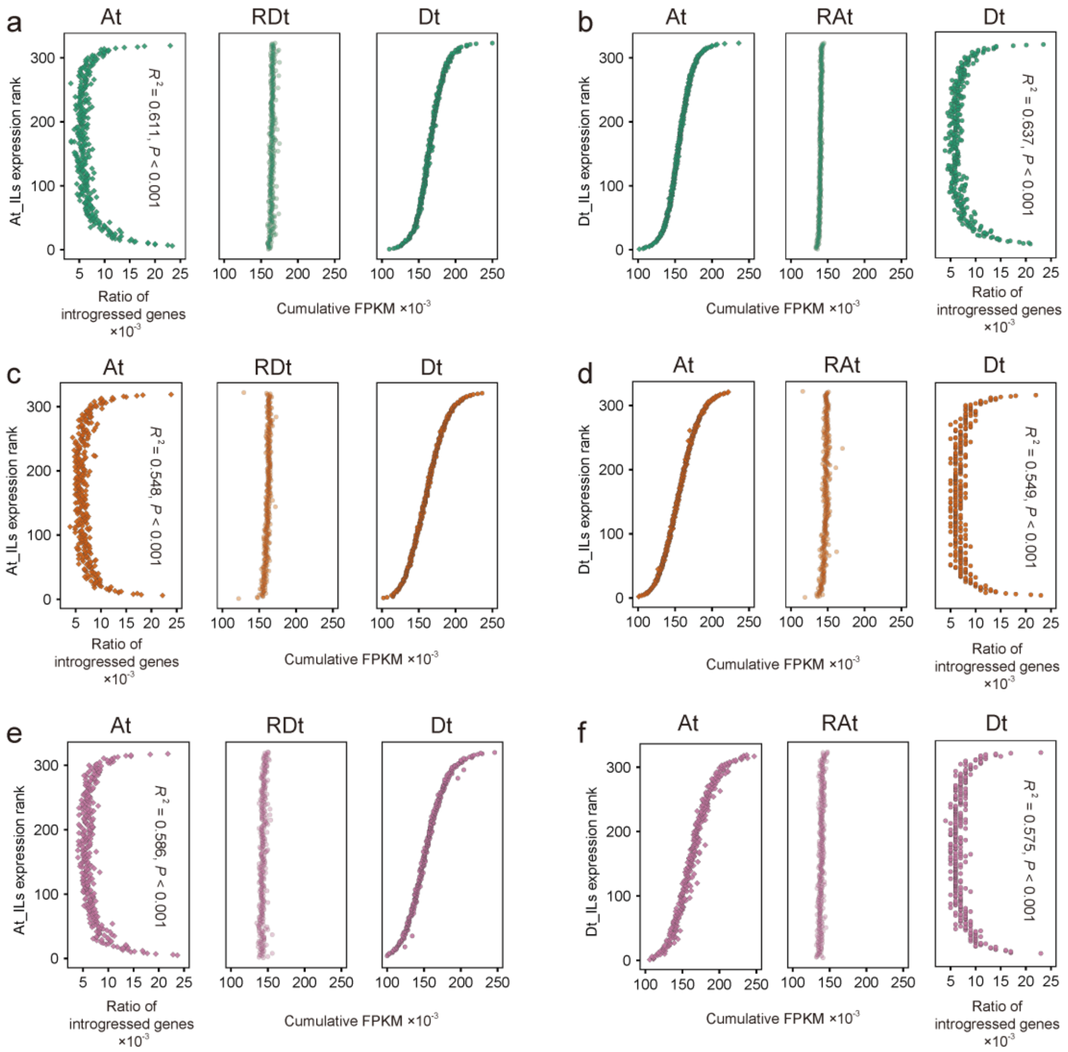

Figure S18 Association of introgressed and non-introgressed copies in homoeologous gene pairs.

Panels **a**, **c** and **e** show the effect of At introgressed copies on Dt non-introgressed copies and random Dt copies in 5 DPA, 10 DPA and 15 DPA. Panels **b**, **d** and **f** show the effect of Dt introgressed copies on At non-introgressed copies and random At copies at 5 DPA, 10 DPA and 15 DPA. Green indicates 5 DPA, orange indicates 10 DPA, and purple indicates 20 DPA.

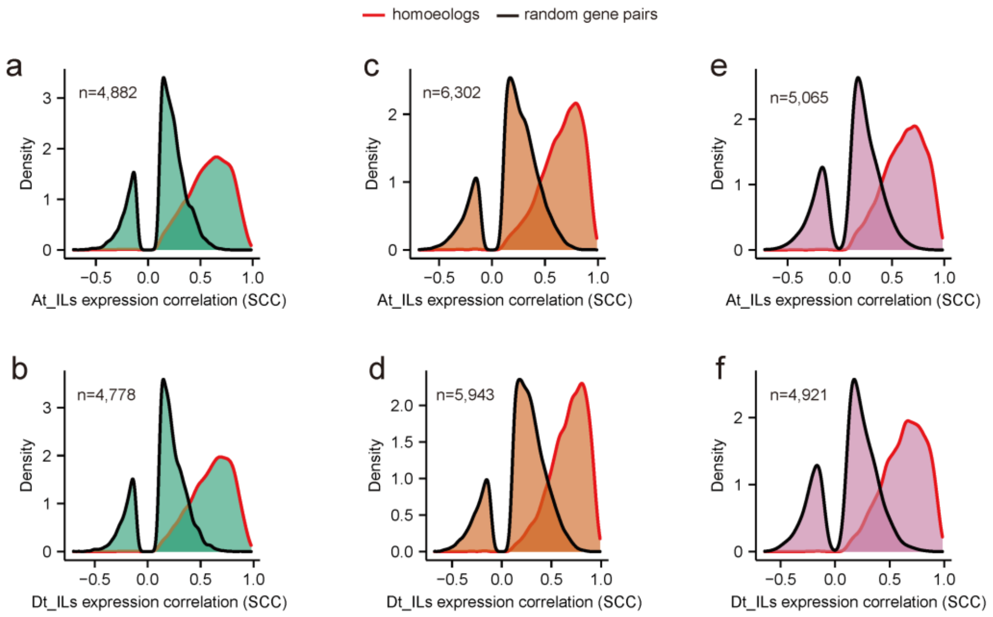

Figure S19 SCC density plots of some homoeologous gene pairs.

Panels **a**, **c** and **e** show the spearman correlation coefficients for homoeologous gene pairs and random gene pairs in introgressed copies of At. Panels **b**, **d** and **f** show the spearman correlation coefficients (SCC) for homoeologous gene pairs and random gene pairs in introgressed copies of Dt.

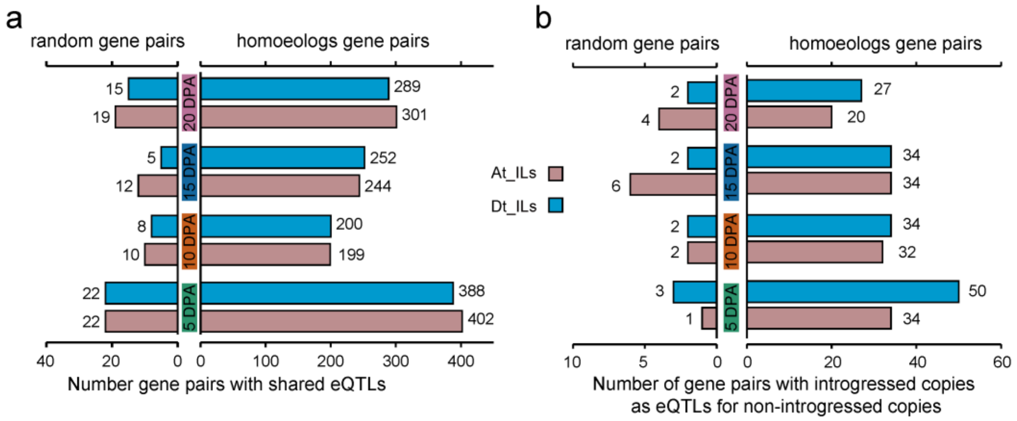

Figure S20 Number of gene pairs with shared eQTLs and introgressed copies as non-introgressed copies of eQTLs in random gene pairs and homoeologous gene pairs.

In **a** and **b**, the left panel of the bar graph represents the number of gene pairs with shared eQTLs/introgressed copies as non-introgressed copies of eQTLs for random gene pairs, and the right panel represents the number of gene pairs with shared eQTLs/introgressed copies as non-introgressed copies of eQTLs for homoeologous gene pairs. Brown (At_ILs) represents the gene pair with introgression for the At copy, and blue (Dt_ILs) represents the gene pair with introgression for the Dt copy.

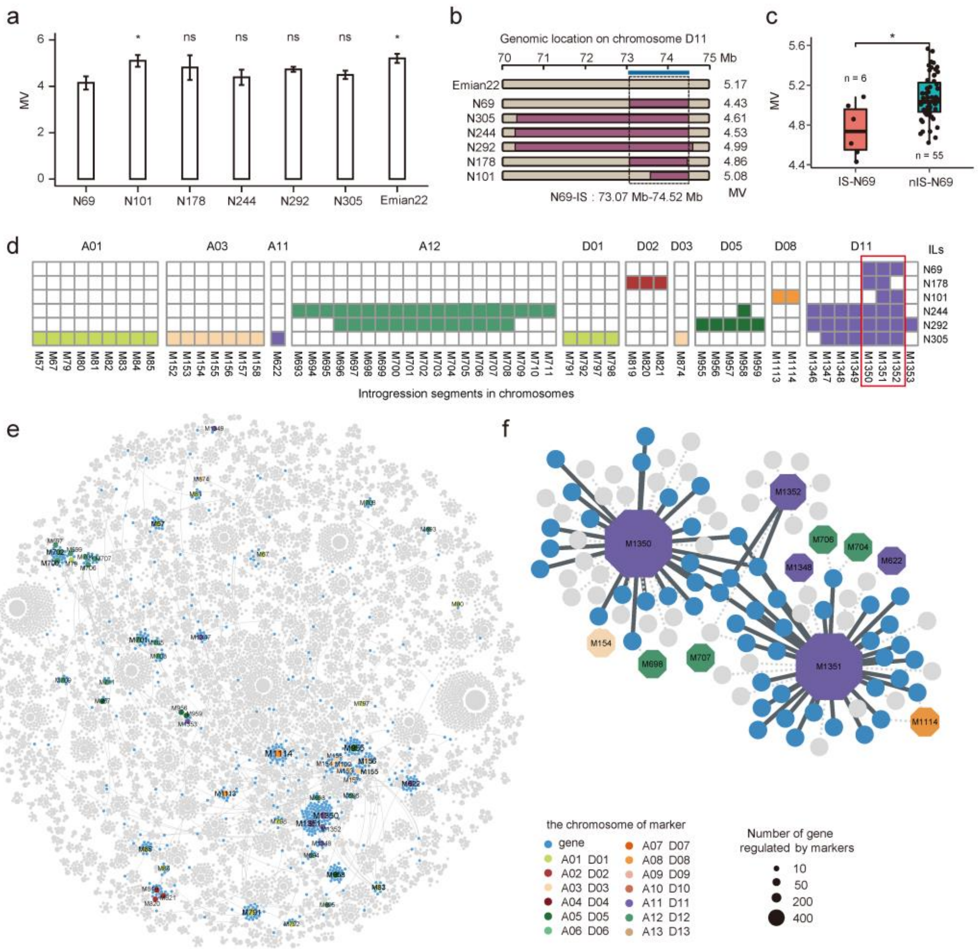

Figure S21 Genetic network of N69-IS and its effect on micronaire value.

a, Significance analysis of N69, Emian22, and other introgression lines carrying N69-IS on micronaire values; n = 5. All significances are tested by two-tailed Student's *t* test. ns, $P > 0.05$; *, $P < 0.05$. Error bars are presented as mean ± SE. **b,** Micronaire values and introgression segment locations for lines containing N69 introgression segments. In panels, purple represents introgression fragments from 3-79, and brown represents fragments from the Emian22 genome. Fiber phenotype values processed by BLUP. **c,** Box plot showing the comparison of micronaire values between accessions that carry N69-IS (n = 6) and those that do not carry N69-IS (n = 55). Center line, median; box limits, first and third quartiles; whisker, 1.5× interquartile range. In all cases, significance is tested by two-tailed Student's *t* test. ns, $P > 0.05$; *, $P < 0.05$. **d,** Genome-wide introgression positions of introgression lines carrying the N69-IS. The x-axis represents the marker ID that each introgression segment is divided into. **e,** Regulatory network of introgression lines carrying N69-IS in Emian22. The introgression segments for each introgression line are marked in the network. Different chromosomes are indicated by the colors described. **f,** Regulatory network of introgression segments in IL N69. Circles indicate eGenes regulated by introgression segments in the network. Blue dots represent regulatory linkages present in IL N69. Polygons represent introgression segments. MV: micronaire value.

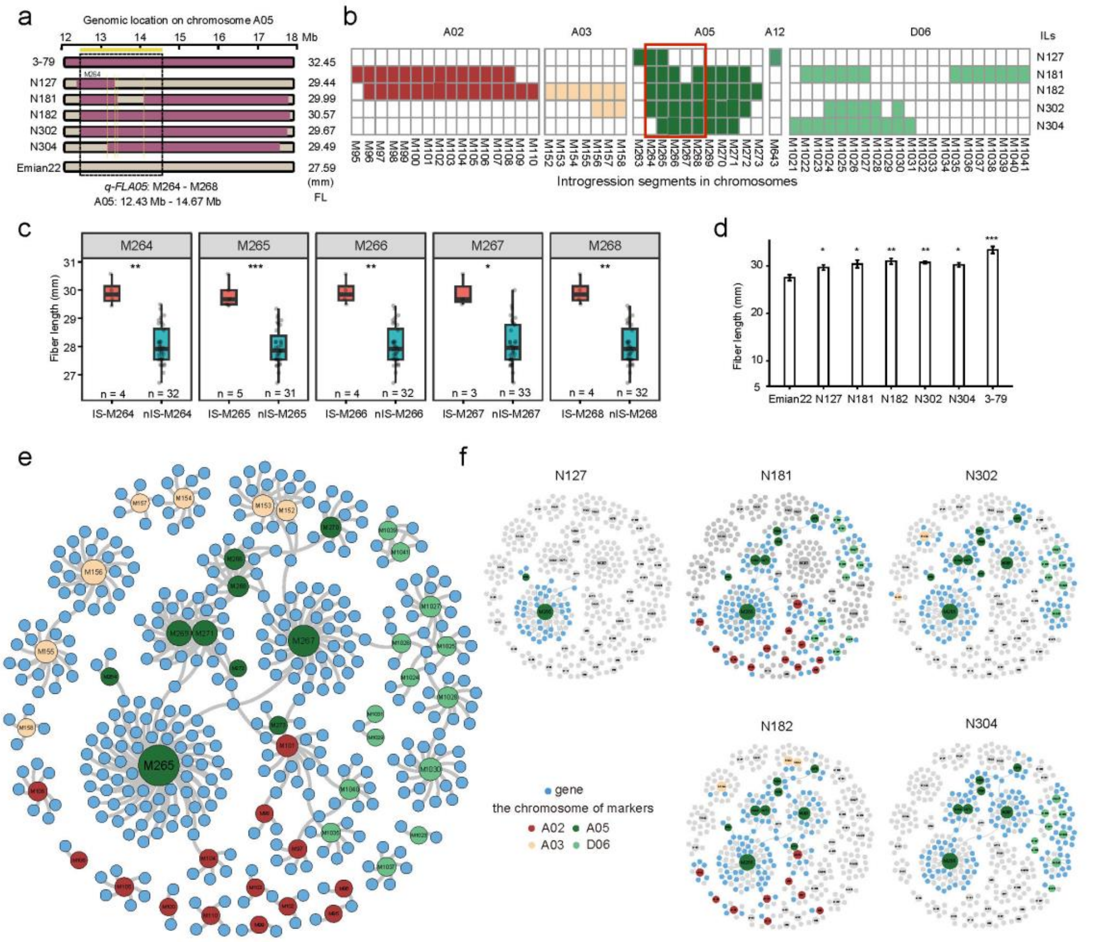

Figure S22 Genetic network of *q-FLA05*-IS and its effect on fiber length.

a, Fiber length and introgression segments locations for lines containing QTLs associated with fiber length on chromosome A05 introgression segments. In panels, purple represents introgression segments from 3-79, and brown represents segments from the Emian22 genome. Fiber phenotype values processed by BLUP. **b,** Genome-wide introgression positions of introgression lines carrying the *q-FLA05*-IS. The x-axis represents the marker ID that each introgression segment is divided into. **c,** Box plot showing the comparison of fiber length between accessions that carry *q-FLA05*-IS and those that do not carry *q-FLA05*-IS. *q-FLA05*-IS contains five QTLs: M264, M265, M266, M267, and M268. Center line, median; box limits, first and third quartiles; whisker, 1.5× interquartile range. In all cases, significance is tested by two-tailed Student's *t* test. ns, $P > 0.05$; *, $P < 0.05$; **, $P < 0.01$; ***, $P < 0.001$. **d,** Significance analysis of Emian22, 3-79 and other introgression lines carrying *q-FLA05*-IS on fiber length; n = 5. All significances are tested by two-tailed Student's *t* test. ns, $P > 0.05$; *, $P < 0.05$; **, $P < 0.01$; ***, $P < 0.001$. Error bars are presented as mean ± SE. **e,** Regulatory network of introgression lines carrying *q-FLA05*-IS in Emian22. The introgression segments for each introgression line are marked in the network. Different chromosomes are indicated by the colors described. **f,** Regulatory network of introgression segments in different ILs. Blue dots indicate eGenes regulated by introgression segments in the network. The links represent eQTL-eGene associations.

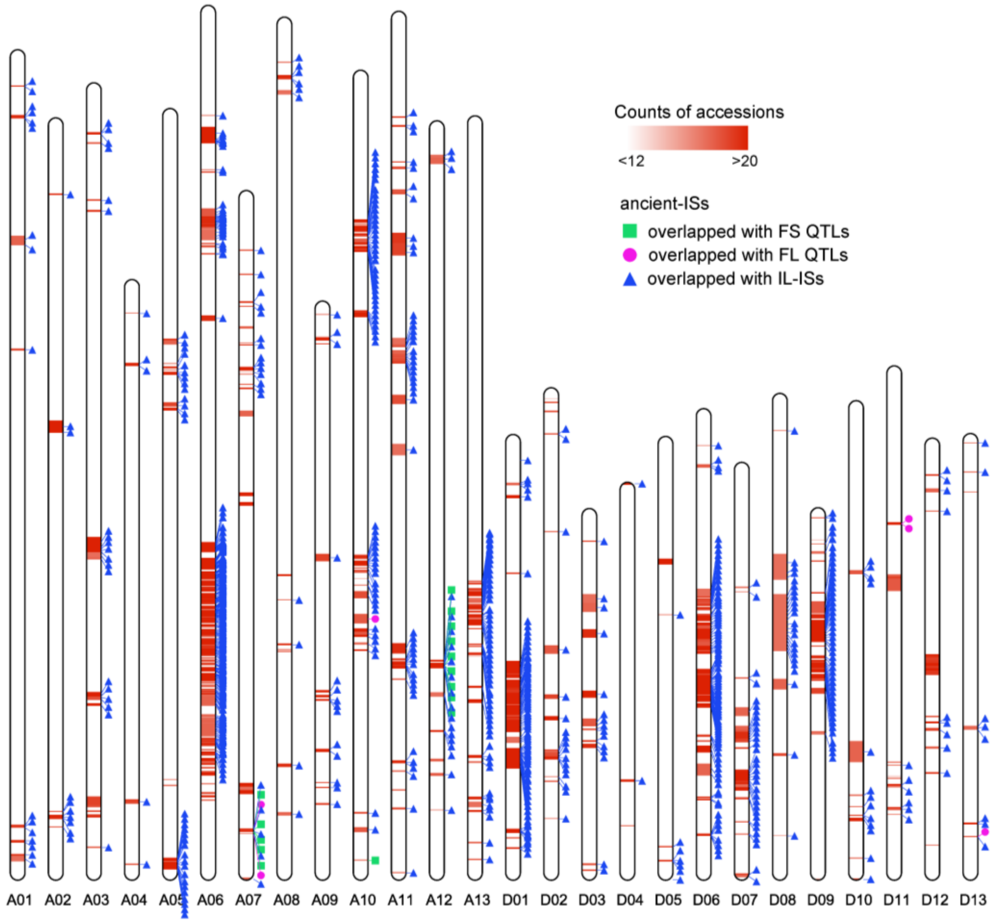

Figure S23 The distribution of ancient introgression segments.

The chromosome level distribution of ancient-ISs. Green box, ancient-ISs overlapped with published FS QTLs; Pink circle, ancient-ISs overlapped with published FL QTLs; Blue triangle, ancient-ISs overlapped with IL-ISs. The heatmap from white to red represents more accessions with introgression segments.

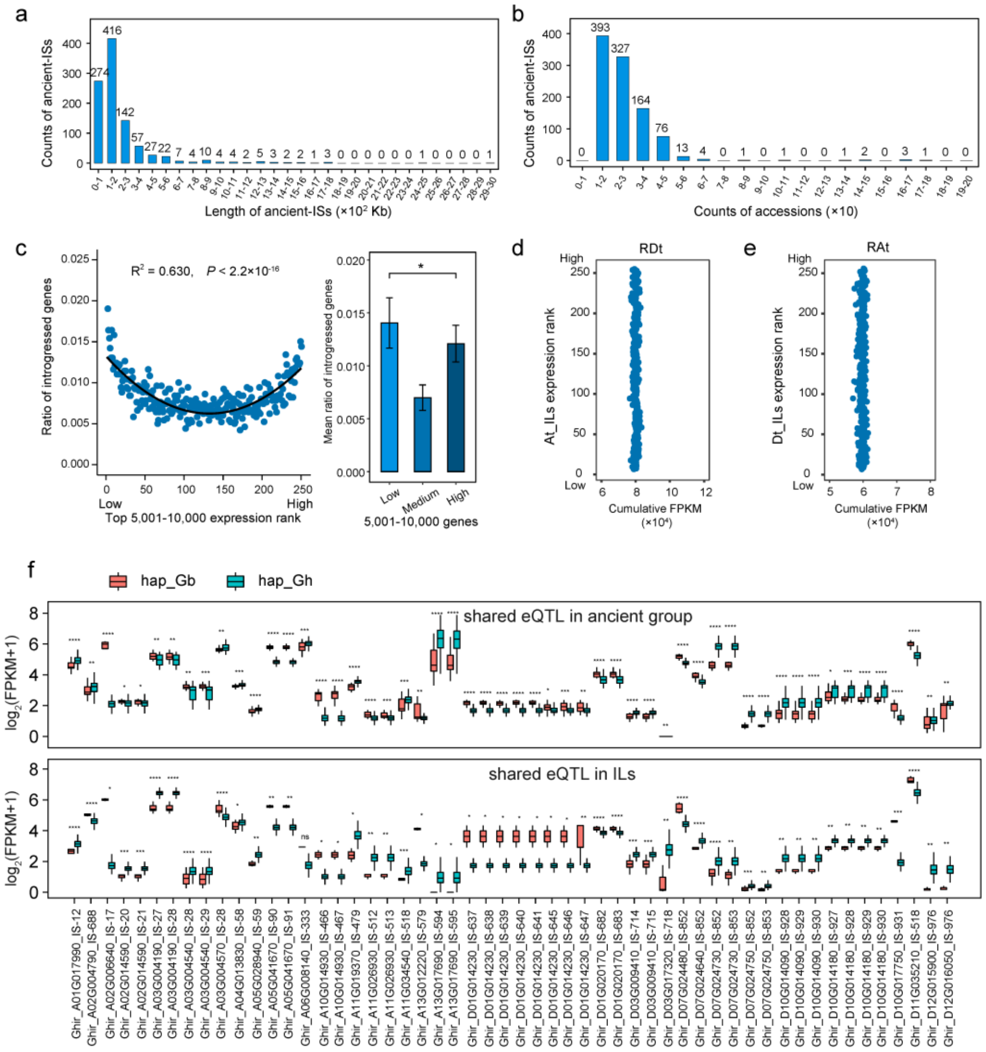

Figure S24 The details of ancient introgression segments.

a, The length distribution of ancient-ISs. **b,** The number of accessions with ancient-ISs. **c,** The relationship between the expression rank with introgression segments. Left panel, dot plot to exhibit the ratio of introgressed genes (y-axis) that exist in corresponding expression rank (x-axis). Right panel, bar plot to exhibit the mean ratio of introgressed genes (y-axis) that exist in corresponding expression rank (x-axis). Low, 0-5% of expression ranks; Medium, 25-75% of expression ranks; High, 95-100% of expression ranks. ****, $P < 0.0001$. Error bars are presented as mean ± SD. **d,** The relationship between the expression rank with introgression segments from At. Dot plot to exhibit the cumulative FPKM of random genes from Dt corresponding to homoeologous genes in At. **e,** The relationship between the expression rank with introgression segments from Dt. Dot plot to exhibit the cumulative FPKM of random genes from At corresponding to homoeologous genes in Dt. **f,** Box plots show 55 shared eQTL-eGene associations between ancient-ISs and IL-ISs. Pink box, haplotypes from *G. barbadense*; Blue box, haplotypes from *G. hirsutum.* ns, $P > 0.05$; *, $P < 0.05$; **, $P < 0.01$; ***, $P < 0.001$; ****, $P < 0.0001$. Center line, median; box limits, first and third quartiles; whisker, 1.5× interquartile range.

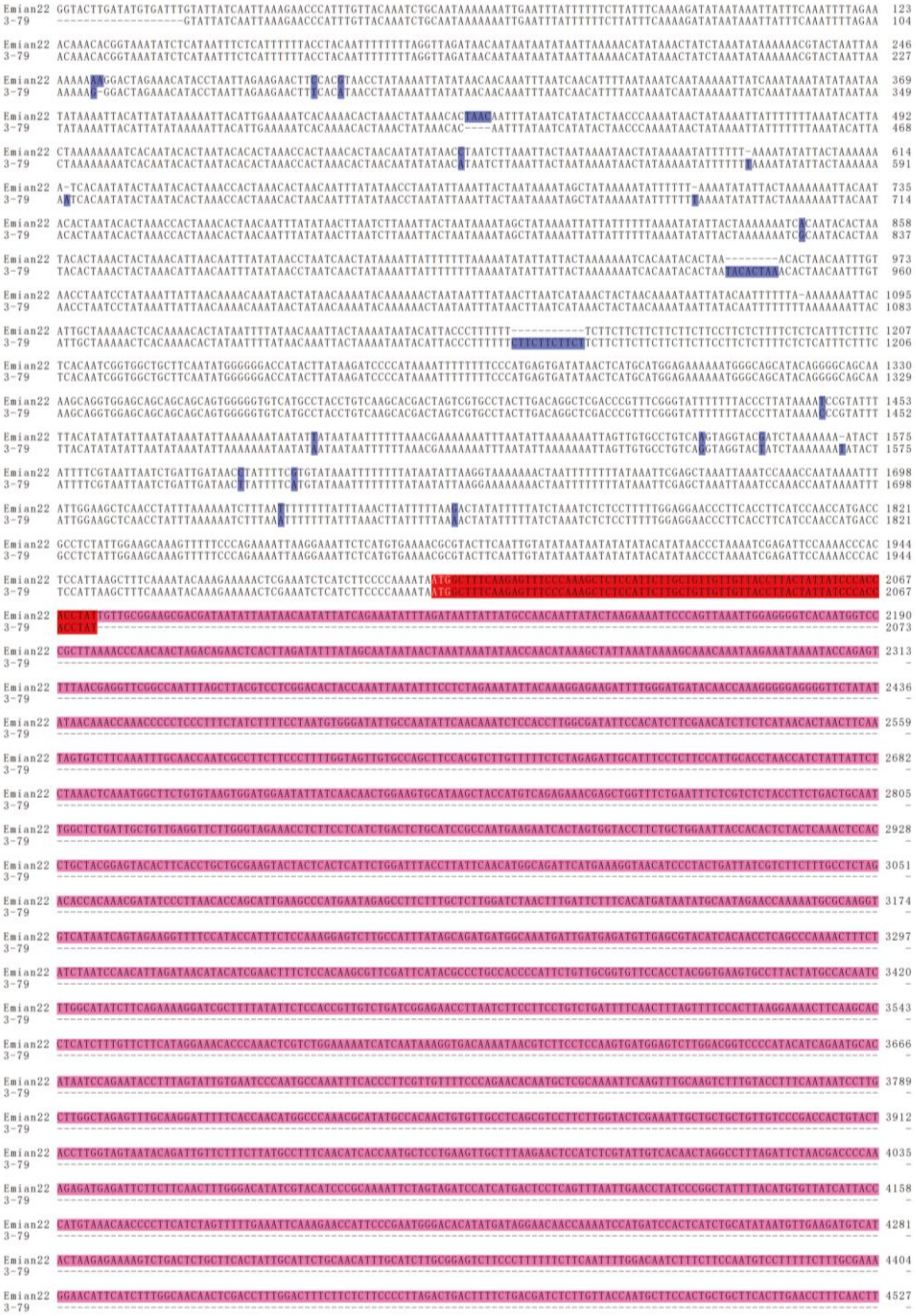

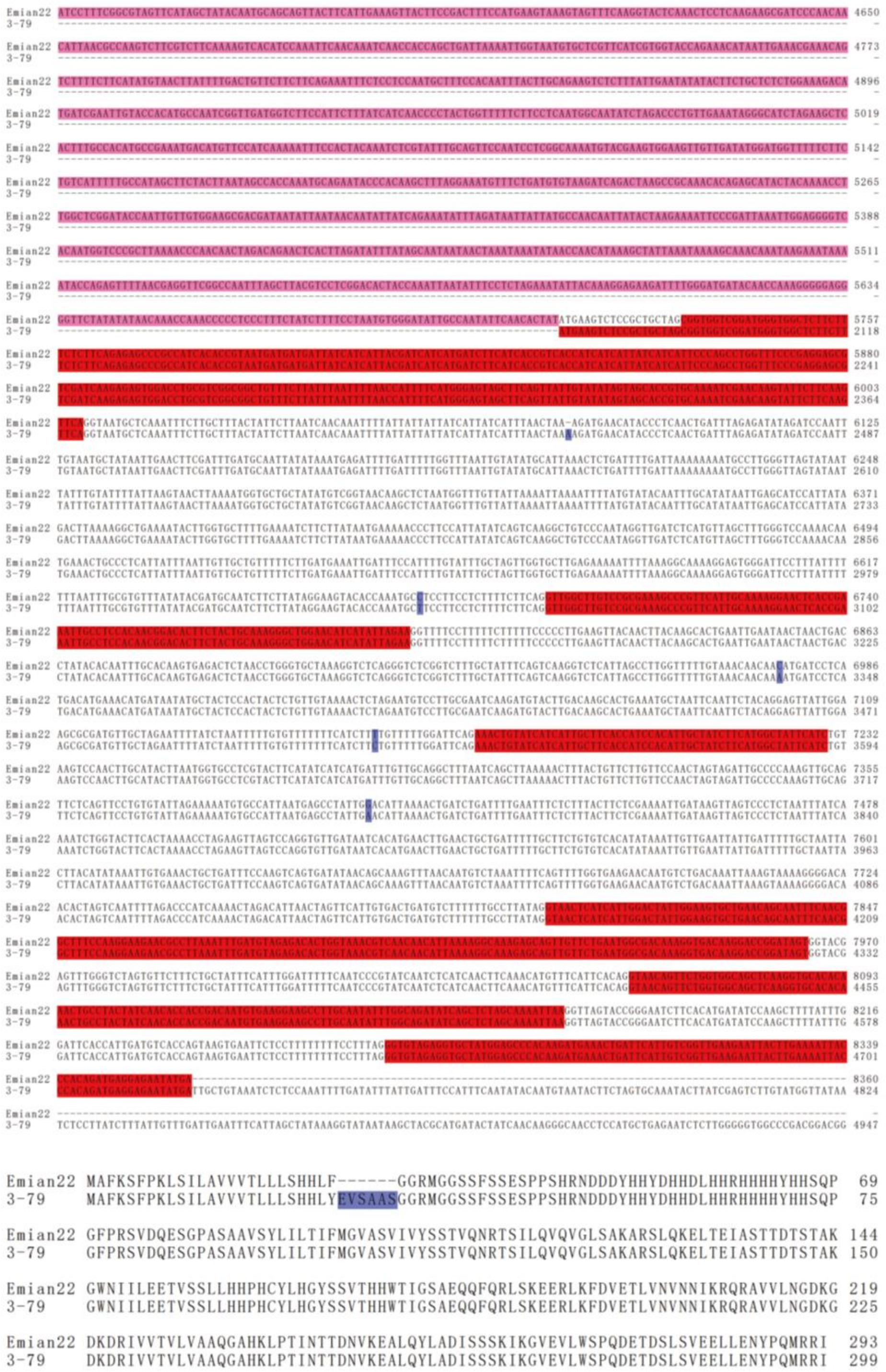

Figure S25 Comparison of *GhFLAP1* allele and protein sequences between Emian22 and 3-79.

The numbers on the right indicate the nucleotide positions in the sequences. Polymorphic sites between Emian22 and 3-79 alleles and proteins are shaded in purple. Exons are shaded in red. The pink shading represents sequence insertion in Emian22 genome.

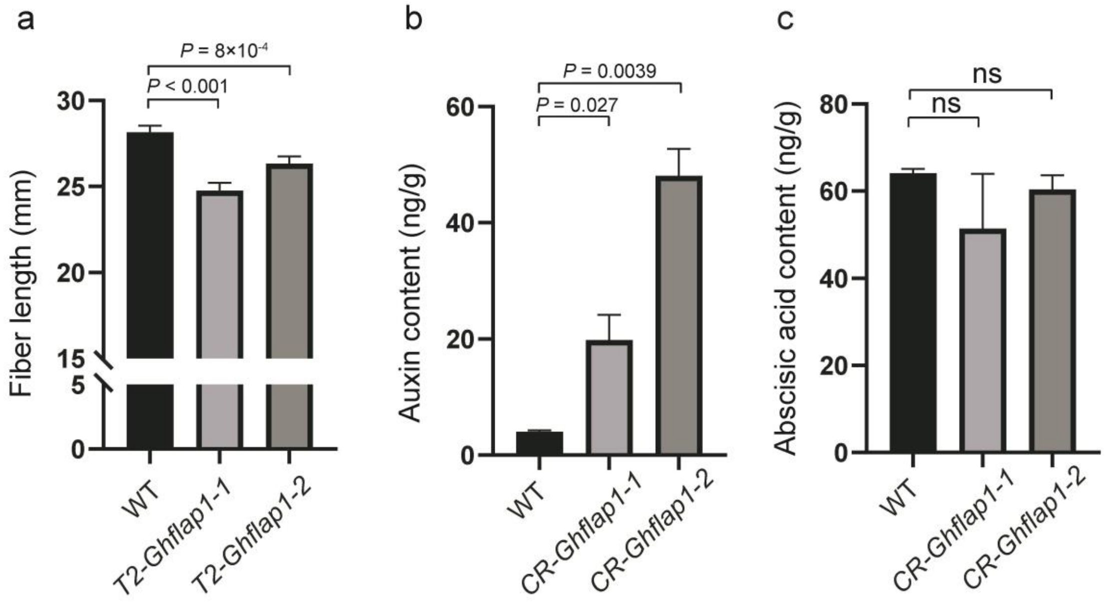

Figure S26 Fiber length and phytohormone content of *Ghflap1* mutants.

a, Fiber length of WT and T2 plants. Statistical significance was derived from Student's *t* test. Error bars are presented as mean ± SD. **b**, Auxin content of *Ghflap1* mutants. Statistical significance was derived from Student's *t* test. Error bars are presented as mean ± SD. **c**, Abscisic acid content of *Ghflap1* mutants. Statistical significance was derived from Student's *t* test, as indicated by ns, $P > 0.05$. Error bars are presented as mean ± SD.

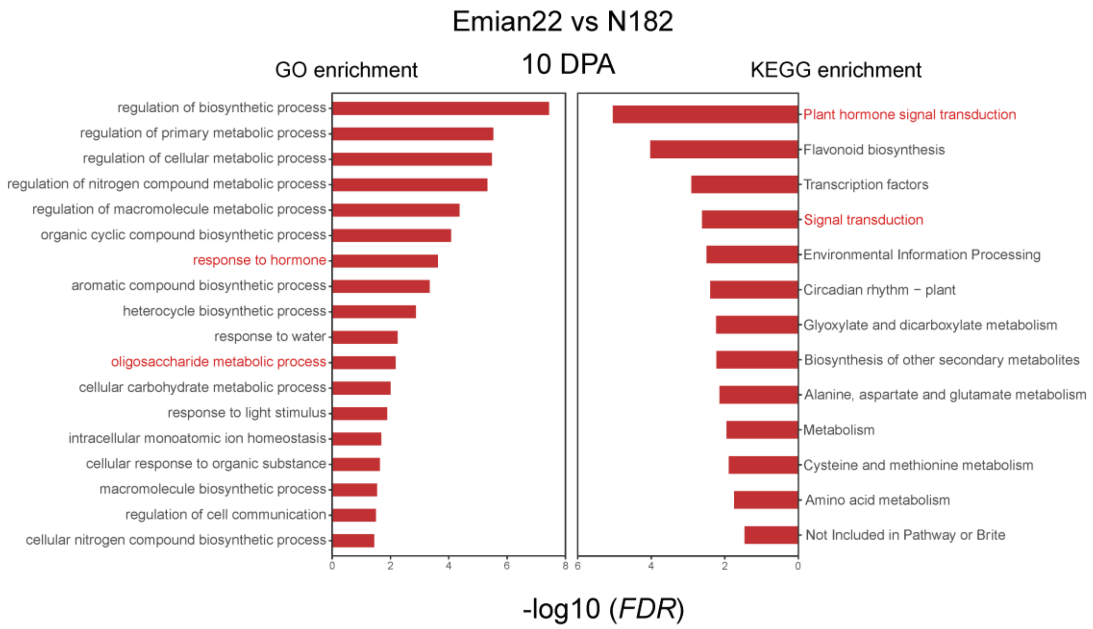

Figure S27 GO and KEGG enrichment analysis of DEGs with up-regulated expression in N182 and down-regulated expression in Emian22 at 10 DPA. *FDR*: false discovery rate.

Supplemental References

[1] Y. Hu, J. D. Chen, L. Fang, Z. Y. Zhang, W. Ma, Y. C. Niu, L. Z. Ju, J. Q. Deng, T. Zhao, J. M. Lian, K. Baruch, D. Fang, X. Liu, Y. L. Ruan, M. U. Rahman, J. L. Han, K. Wang, Q. Wang, H. T. Wu, G. F. Mei, Y. H. Zang, Z. G. Han, C. Y. Xu, W. J. Shen, D. F. Yang, Z. F. Si, F. Dai, L. F. Zou, F. Huang, Y. L. Bai, Y. G. Zhang, A. Brodt, H. Ben-Hamo, X. F. Zhu, B. L. Zhou, X. Y. Guan, S. J. Zhu, X. Y. Chen, T. Z. Zhang, *Nat. Genet.* **2019**, *51* (4), 739, https://doi.org/10.1038/s41588-019-0371-5.

[2] H. Li, *arXiv preprint arXiv:1303.3997* **2013**.

[3] H. Li, B. Handsaker, A. Wysoker, T. Fennell, J. Ruan, N. Homer, G. Marth, G. Abecasis, R. Durbin, S. Genome Project Data Processing, *Bioinformatics* **2009**, *25* (16), 2078, https://doi.org/10.1093/bioinformatics/btp352.

[4] C. Zang, D. E. Schones, C. Zeng, K. Cui, K. Zhao, W. Peng, *Bioinformatics* **2009**, *25* (15), 1952.

[5] H. Li, *Bioinformatics* **2018**, *34* (18), 3094, https://doi.org/10.1093/bioinformatics/bty191.

[6] Z. Yang, X. Ge, Z. Yang, W. Qin, G. Sun, Z. Wang, Z. Li, J. Liu, J. Wu, Y. Wang, L. Lu, P. Wang, H. Mo, X. Zhang, F. Li, *Nat. Commun.* **2019**, *10* (1), 2989, https://doi.org/10.1038/s41467-019-10820-x.

[7] Z. Ma, Y. Zhang, L. Wu, G. Zhang, Z. Sun, Z. Li, Y. Jiang, H. Ke, B. Chen, Z. Liu, Q. Gu, Z. Wang, G. Wang, J. Yang, J. Wu, Y. Yan, C. Meng, L. Li, X. Li, S. Mo, N. Wu, L. Ma, L. Chen, M. Zhang, A. Si, Z. Yang, N. Wang, L. Wu, D. Zhang, Y. Cui, J. Cui, X. Lv, Y. Li, R. Shi, Y. Duan, S. Tian, X. Wang, *Nat. Genet.* **2021**, *53* (9), 1385, https://doi.org/10.1038/s41588-021-00910-2.

[8] G. Huang, Z. G. Wu, R. G. Percy, M. Z. Bai, Y. Li, J. E. Frelichowski, J. Hu, K. Wang, J. Z. Yu, Y. X. Zhu, *Nat. Genet.* **2020**, *52* (5), 516, https://doi.org/10.1038/s41588-020-0607-4.

[9] Z. J. Chen, A. Sreedasyam, A. Ando, Q. X. Song, L. M. De Santiago, A. M. Hulse-Kemp, M. Q. Ding, W. X. Ye, R. C. Kirkbride, J. Jenkins, C. Plott, J. Lovell, Y. M. Lin, R. Vaughn, B. Liu, S. Simpson, B. E. Scheffler, L. Wen, C. A. Saski, C. E.

Grover, G. J. Hu, J. L. Conover, J. W. Carlson, S. Q. Shu, L. B. Boston, M. Williams, D. G. Peterson, K. McGee, D. C. Jones, J. F. Wendel, D. M. Stelly, J. Grimwood, J. Schmutz, *Nat. Genet.* **2020**, *52* (5), 525, https://doi.org/10.1038/s41588-020-0614-5.

[10] M. Wang, L. Tu, D. Yuan, D. Zhu, C. Shen, J. Li, F. Liu, L. Pei, P. Wang, G. Zhao, Z. Ye, H. Huang, F. Yan, Y. Ma, L. Zhang, M. Liu, J. You, Y. Yang, Z. Liu, F. Huang, B. Li, P. Qiu, Q. Zhang, L. Zhu, S. Jin, X. Yang, L. Min, G. Li, L. L. Chen, H. Zheng, K. Lindsey, Z. Lin, J. A. Udall, X. Zhang, *Nat. Genet.* **2019**, *51* (2), 224, https://doi.org/10.1038/s41588-018-0282-x.
